# Cobaloximes as Building Blocks in Halogen-Bonded Cocrystals

**DOI:** 10.3390/ma13102370

**Published:** 2020-05-21

**Authors:** Nikola Bedeković, Valentina Martinez, Edi Topić, Vladimir Stilinović, Dominik Cinčić

**Affiliations:** 1 Department of Chemistry, Faculty of Science, University of Zagreb, Horvatovac 102A, 10 000 Zagreb, Croatia; nbedekovic@chem.pmf.hr (N.B.); vmartin@irb.hr (V.M.); edi.topic@chem.pmf.hr (E.T.); 2 Laboratory for Green Synthesis, Ruđer Bošković Institute, Bijenička cesta 54, 10 000 Zagreb, Croatia

**Keywords:** halogen bonding, cobaloximes, coordination compounds, cocrystals, mechanochemistry

## Abstract

In this work, we explore the halogen-bonded cocrystallization potential of cobaloxime complexes in the synthesis of cocrystals with perhalogenated benzenes. We demonstrate a strategy for synthesizing halogen-bonded metal–organic cocrystals by utilizing cobaloximes whose pendant bromide group and oxime oxygen enable halogen bonding. By combining three well-known halogen bond donor molecules differing in binding geometry and composition with three cobaloxime units, we obtained a total of four previously unreported cocrystals. Single crystal X-ray diffraction experiments showed that the majority of obtained cocrystals exhibited the formation of the targeted I···O and I···Br motives. These results illustrate the potential of cobaloximes as halogen bond acceptors and indicate that this type of halogen bond acceptors may offer a novel route to metal–organic halogen-bonded cocrystals.

## 1. Introduction

Since the revival of research into halogen bonding [1,2,3], there have been two main avenues of research: the fundamental study of the nature of halogen bonding, and the application of halogen bonding in the design of new materials. The results of the former (such as the predominantly electrostatic [4] and occasionally covalent [5,6,7,8] nature of the interaction, comparison with hydrogen bonding [9,10,11], the possibility of bifurcation [12,13,14], and metal and metalloid atoms as acceptors [15,16]) have contributed to a considerable extension of the possibilities for preparing various halogen-bonded functional materials [17,18], such as liquid crystals [19,20], anion receptors [21,22], photoluminescent [23,24] and photoreactive materials, and gelators [25,26,27].

The majority of research into halogen-bonded materials, however, has been dealing with purely organic materials—both halogen donor and acceptor being organic molecules. As introducing metal atoms into a supramolecular material can significantly expand the possibilities of adding and controlling additional properties (such as magnetism and conductivity) [28,29], the use of halogen bonding in the crystal engineering of metal–organic materials has been an attractive challenge over the past decade [30,31,32,33,34,35,36,37,38,39,40]. While there have been substantial studies into halogen bonding in single component metal–organic solids [30,31,32,33,34,35,36,37,38,39,40,41], synthesis of multi-component materials based on halogen-bonded metal–organic components has been receiving much less attention. Particularly scarce are studies involving cocrystals of neutral metal–organic components acting as halogen bond acceptors [42,43,44,45,46,47]. The most promising building blocks to date have been metal coordination complexes comprised of pendant acceptor groups such as halogenide ligands [46,47], chelating ligands such as imines [43], β-dicarbonyl compounds [14] and ligands with additional (non-coordinating) electron rich atoms or groups such as morpholine or thiomorpholine [48,49], which can form cocrystals with halogen bond donors, such as perfluorinated iodobenzenes.

**Scheme 1:**
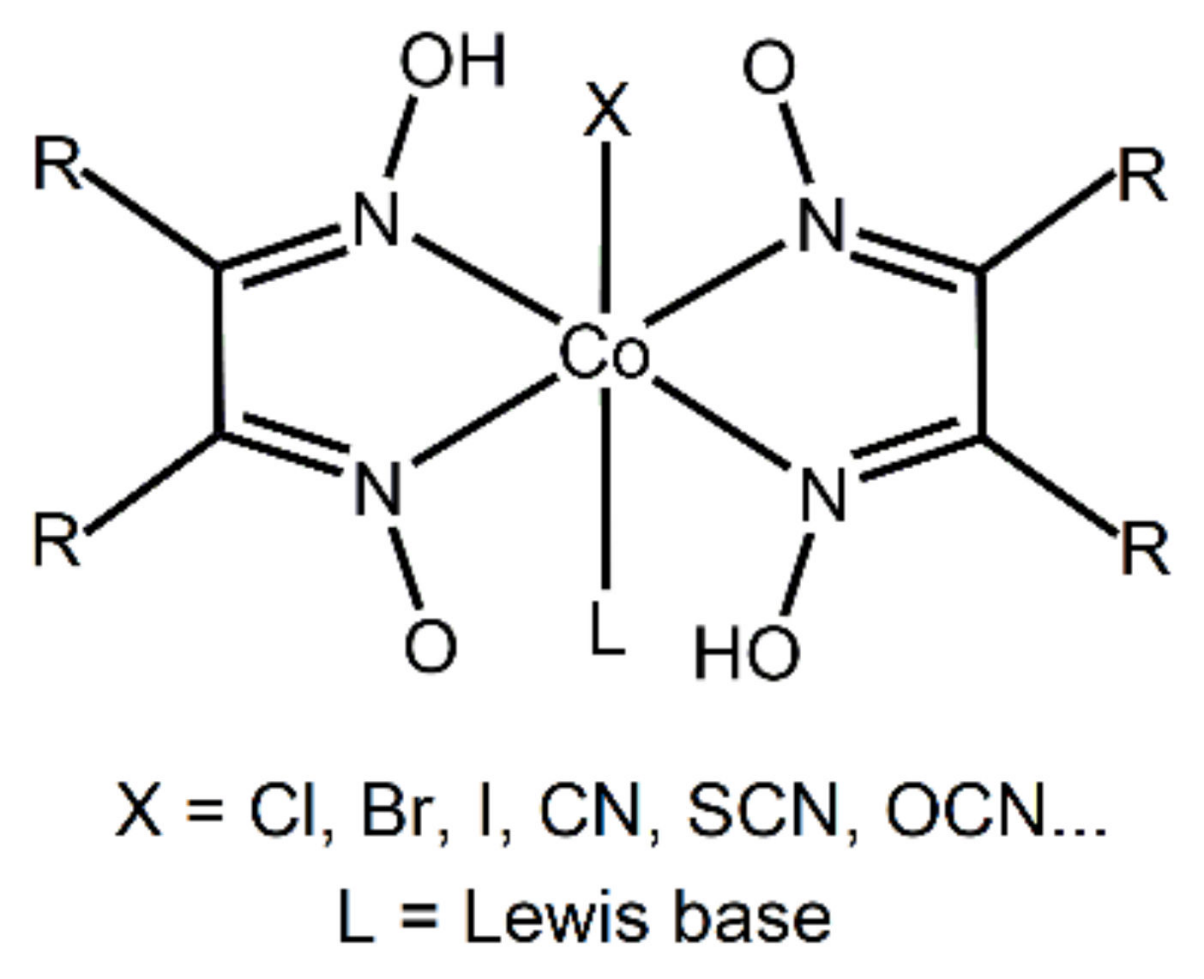
General structure of cobaloximes.

A potentially interesting series of targets for synthesizing such cocrystals are cobaloximes (Scheme 1). These compounds were originally studied as analogues of cobalamine (vitamin B12) [50,51], but more recently have been drawing considerable attention as starting materials for the formation of organocobalt compounds [52,53], as well as catalysts for low-energy production of hydrogen [53,54,55]. As they can be modified by the addition of various substituents on the pyridine ring, as well as by using different anionic ligands (X), they represent a vast and versatile group of compounds with tunable structures and properties. A cursory search of the Cambridge Structural Database (CSD) [56] based on oxime complexes of any transition metal resulted in 1521 hits; 191 of those are for structures of the MX(ox)_2_ type (X = halogen atom; ox = any oxime ligand). Restraining the search further, to Co complexes, resulted in 152 hits. Of those, there are no entries corresponding to halogen-bonded cocrystals, although there has been a report of halogen bonding in single-component crystals of cobaloximes with bromopyridine ligands [57]. Furthermore, a survey based on the ability of any transition metaloxime complex to act as a halogen bond acceptor with haloperfluorinated benzenes as halogen bond donors resulted in no hits.

**Scheme 2:**
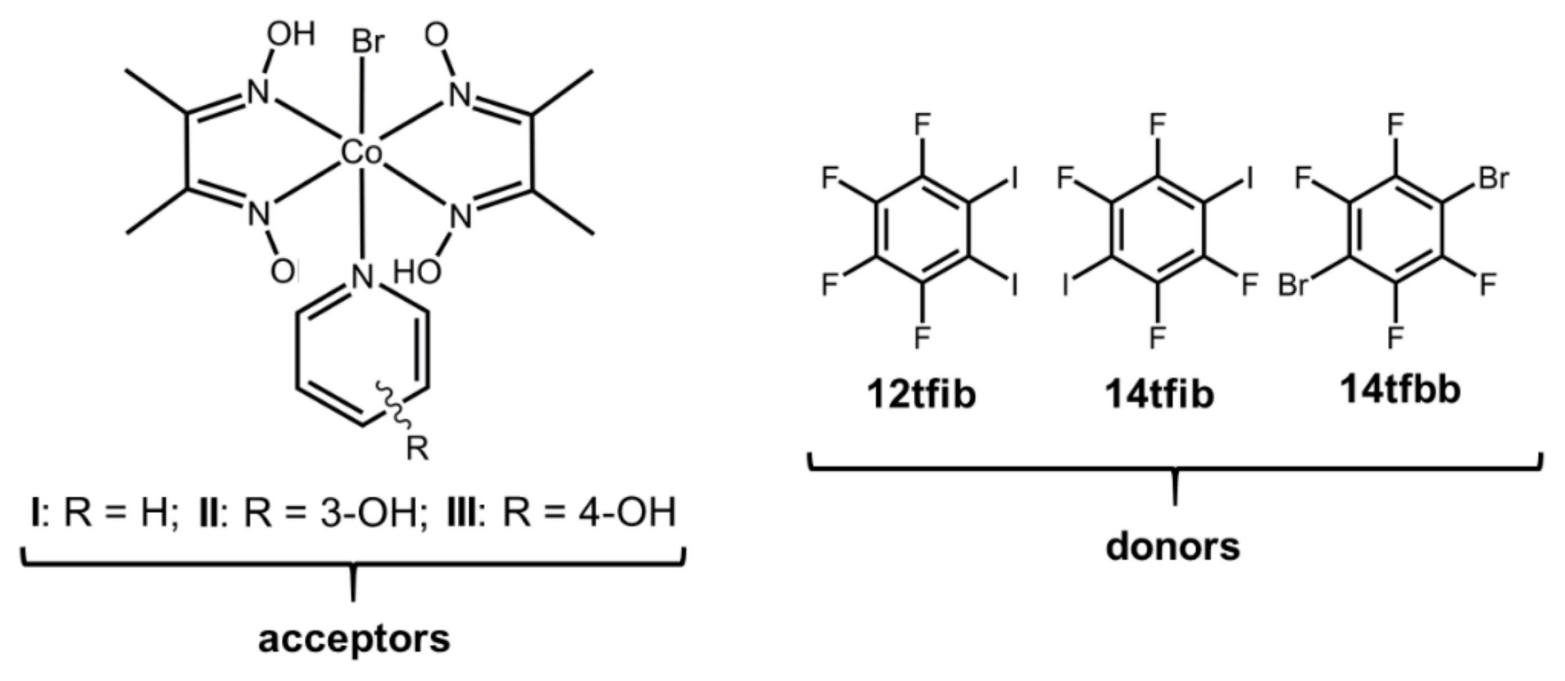
Halogen bond donors and acceptors used in this study.

In this work, we explored the ability to form halogen-bonded cocrystals with cobaloximes as halogen bond acceptors. For this purpose, we prepared three different cobaloxime derivatives (Scheme 2), which contain a pair of dimethylglyoximate anions, and one bromide anion coordinated to the metal center, and different pyridine ligands bonded to the axial coordination site. Their pendant and different functional groups enable halogen bonding: oxygen atoms on the oxime ligand, the coordinated bromine atom, as well as the π-system and the hydroxyl substituents of the pyridine ring can all act as halogen bond acceptor sites. For cocrystal synthesis, as halogen bond donors, we selected a series of perhalogenated benzenes that differ in the number and positions of donor atoms: 1,2-diiodotetrafluorobenzene (**12tfib**), 1,4-diiodotetrafluorobenzene (**14tfib**) and 1,4-dibromotetrafluorobenzene (**14tfbb**), Scheme 1. Additionally, we wanted to observe whether the additional hydrogen bond donor functionality introduced through the hydroxypyridine ligands would hinder the formation of the desired halogen bond. Thus, we prepared three cobaloxime complexes. Complex **I** contained an unsubstituted pyridine molecule that does not contain any functional groups prone to creating additional noncovalent interactions. On the other hand, 3- and 4-hydroxypyridine were present in the complexes **II** and **III**, respectively, which may act as hydrogen bond donors and form a wide range of disparate supramolecular motifs. The introduction of a hydroxyl group on the pyridine also affects the hydrogen (and halogen) bond acceptor potential of the metal complex molecule: in **I** the available acceptor sites are the four oxime oxygen atoms and the bromide ligand, while **II** and **III** can additionally form bonds via the hydroxyl oxygen atoms.

## 2. Materials and Methods

### 2.1. Synthesis of Metal Complexes

Metal complexes (Figures S1 and S2 in Supplementary Materials) were prepared as follows: cobalt(II) acetate dihydrate (Sigma-Aldrich Chemie GmbH, Taufkirchen, Germany) (0.82 g) was dissolved in hot ethanol (Sigma-Aldrich Chemie GmbH, Taufkirchen, Germany) (4.0 mL), and then concentrated hydrobromic acid (Sigma-Aldrich Chemie GmbH, Taufkirchen, Germany) was added (380 μL)—solution A. Dimethylglyoxime (Sigma-Aldrich Chemie GmbH, Taufkirchen, Germany) (H**dmg**; 0.78 g) and 3-hydroxypyridine or 4-hydroxypyridine (Sigma-Aldrich Chemie GmbH, Taufkirchen, Germany) (0.32 g) were dissolved in hot ethanol (Alkaloid, Skopje, Republic of North Macedonia) (10 mL), and afterwards, triethylamine (Sigma-Aldrich Chemie GmbH, Taufkirchen, Germany) was added (1.0 mL)—solution B. Solutions A and B were mixed in a test tube, and the resulting solution was oxidized by passing air through the mixture. After several hours, brown precipitates of the corresponding cobaloxime complexes formed and were then filtered off. Single crystals of **I** and **II** were obtained after dissolving 30 mg of complex in 2.0 mL of a hot mixture of tetrahydrofuran (Sigma-Aldrich Chemie GmbH, Taufkirchen, Germany) and water (Zagrebački holding, Zagreb, Croatia) (volume ratio 3:1) and the subsequent cooling and solvent evaporation at room temperature for 2 days. All crystallization experiments for **III** resulted in crystals that were not suitable for the X-ray diffraction experiment.

### 2.2. Synthesis of Cocrystals

In order to examine the ability of the prepared compounds **I**, **II** and **III** to form cocrystals with halogen bond donors (**12tfib**, **14tfib,** and **14tfbb**), the obtained complexes and donors were ground in 1:1 and 2:1 stoichiometric ratios, with the addition of small amounts of nitromethane (Sigma-Aldrich Chemie GmbH, Taufkirchen, Germany) (20 μL). The obtained powders were then characterized by XRPD to test whether a new phase had formed. The grinding experiments were conducted in a Retsch MM200 ball mill (Retsch, Haan, Germany) using 10 mL stainless steel jars and two stainless steel balls (5 mm in diameter) for 30 min, under normal laboratory conditions (40–60% relative humidity and temperature *ca*. 25 °C). Masses and volumes of the reactants used in the mechanochemical synthesis of cocrystals are given in Table S1 and Table S2 in the Supplementary Materials.

Single crystals of compounds whose formation was confirmed by the mechanochemical experiments were prepared by crystallization from the solution. The corresponding halogen bond donors and metal–organic complexes were dissolved in 3.0 mL of a hot mixture of tetrahydrofuran and acetonitrile (volume ratio 2:1), whereupon the solutions were left to cool and evaporate (experimental details are given in the Supplementary Materials). Crystals suitable for single crystal X-ray diffraction experiments appeared after three to five days. ORTEP plots of the obtained compounds are shown in Figures S3–S6 in Supplementary Materials.

### 2.3. X-Ray Diffraction Measurements

Single crystal X-ray diffraction experiments were performed using an Oxford Diffraction Xcalibur Kappa CCD X-ray diffractometer (Oxford Diffraction Ltd., Abingdon, UK) with graphite-monochromated Mo*K*_α_ (*λ* = 0.71073 Å) radiation. The data sets were collected using the *ω*-scan mode over the 2*θ*—range up to 54°. Programs CrysAlis CCD and CrysAlis RED were employed for data collection, cell refinement, and data reduction [58,59]. The structures were solved by direct methods and refined using the SHELXS (Version 2013, Göttingen, Germany) and SHELXL programs (Version 2013, Göttingen, Germany), respectively [60,61]. The structural refinement was performed on *F*^2^ using all data. The hydrogen atoms were placed in calculated positions and treated as riding on their parent atoms [C–H = 0.93 Å and *U*_iso_(H) = 1.2 *U*_eq_(C); C–H = 0.97 Å and *U*_iso_(H) = 1.2 *U*_eq_(C)]. All calculations were performed using the WinGX crystallographic suite of programs [62]. The figures were prepared using Mercury (Version 4.3.1., CCDC, Cambridge, UK) [63]. Crystallographic data of the prepared compounds are shown in Table S3 in Supplementary Materials.

Powder X-ray diffraction (XRPD) experiments on the samples were performed on a PHILIPS PW 1840 X-ray diffractometer (Philips Analytical, Almelo, The Netherlands) with Cu*K*_α1_ (1.54056 Å) radiation at 40 mA and 40 kV. The scattered intensities were measured with a scintillation counter. The angular range was from 5° to 45° (2*θ*) with a continuous step size of 0.02° and measuring a time of 0.5 s per step. Data collection was performed using the program package Philips X’Pert (Version 1.3e, Philips Analytical, Almelo, The Netherlands) [64] and analysed in X’Pert HighScore Plus (Version 2.2, Malvern Panalytical, Malvern Worcestershire, UK) [65]. Comparisons of the measured and calculated diffractograms for cocrystals are shown in Figures S7–S13 in the Supplementary Materials).

### 2.4. Thermal Analysis

Differential scanning calorimetry (DSC) measurements were performed on a Mettler-Toledo DSC823e module (Mettler Toledo, Greifensee, Switzerland) in sealed aluminium pans (40 μL) with three pinholes in the lid, heated in a stream of nitrogen (150 mL·min^−1^) at a heating rate of 10 °C·min^−1^.

Thermogravimetric measurements (TG) were performed on a Metler-Toledo TGA/SDTA 851e module (Mettler Toledo, Greifensee, Switzerland). Samples were placed in sealed 40 μL aluminium pans with three pinholes and heated from 25 to 500 °C, at a rate of 10 °C·min^−1^ under nitrogen flow of 150 mL·min^−1^.

Data collection and analysis were performed using the program package STARe Software (Version 15.00, Mettler Toledo, Greifensee, Switzerland) [66]. TG and DSC thermograms of the prepared compounds are shown in Figures S14–S25 in Supplementary Materials.

### 2.5. Calculations

Hirshfeld surfaces and fingerprint plots were calculated using CrystalExplorer [67] (Version 3.1., University of Western Australia, Crawley, Australia). Hirshfeld surfaces and fingerprint plots are shown in Figures S26–S31 in Supplementary Materials.

## 3. Results and Discussion

### 3.1. Syntheses of Cocrystals

The large difference in solubility between the used metal–organic complexes and halogen bond donors in most organic solvents created a considerable challenge in the synthesis of cocrystals by crystallization from the solution. Mechanochemical synthesis stood out as a better method for the preparation of this class of compounds, since dissolving the reactants was not a prerequisite for successful cocrystal synthesis. Screening experiments have shown that five out of a total of nine reactant combinations resulted in the formation of new crystalline products, as evidenced by the appearance of new Bragg reflections in the PXRD patterns upon milling (Figures S7–S13). Grinding experiments of the complex **I** and halogen bond donors have resulted in cocrystal formation only with **12tfib**. Furthermore, a total of four new cocrystals of complexes **II** and **III** were prepared with linear halogen bond donors **14tfib** and **14tfbb**. Subsequent single crystal X-ray diffraction experiments on samples grown from the solution have shown that four products are halogen-bonded cocrystals of the following compositions: (**I**)(**12tfib**), (**II**)_2_(**14tfib**), (**III**)_2_(**14tfib**) and (**III**)_2_(**14tfbb**).

### 3.2. Structural Analysis

**Figure 1:**
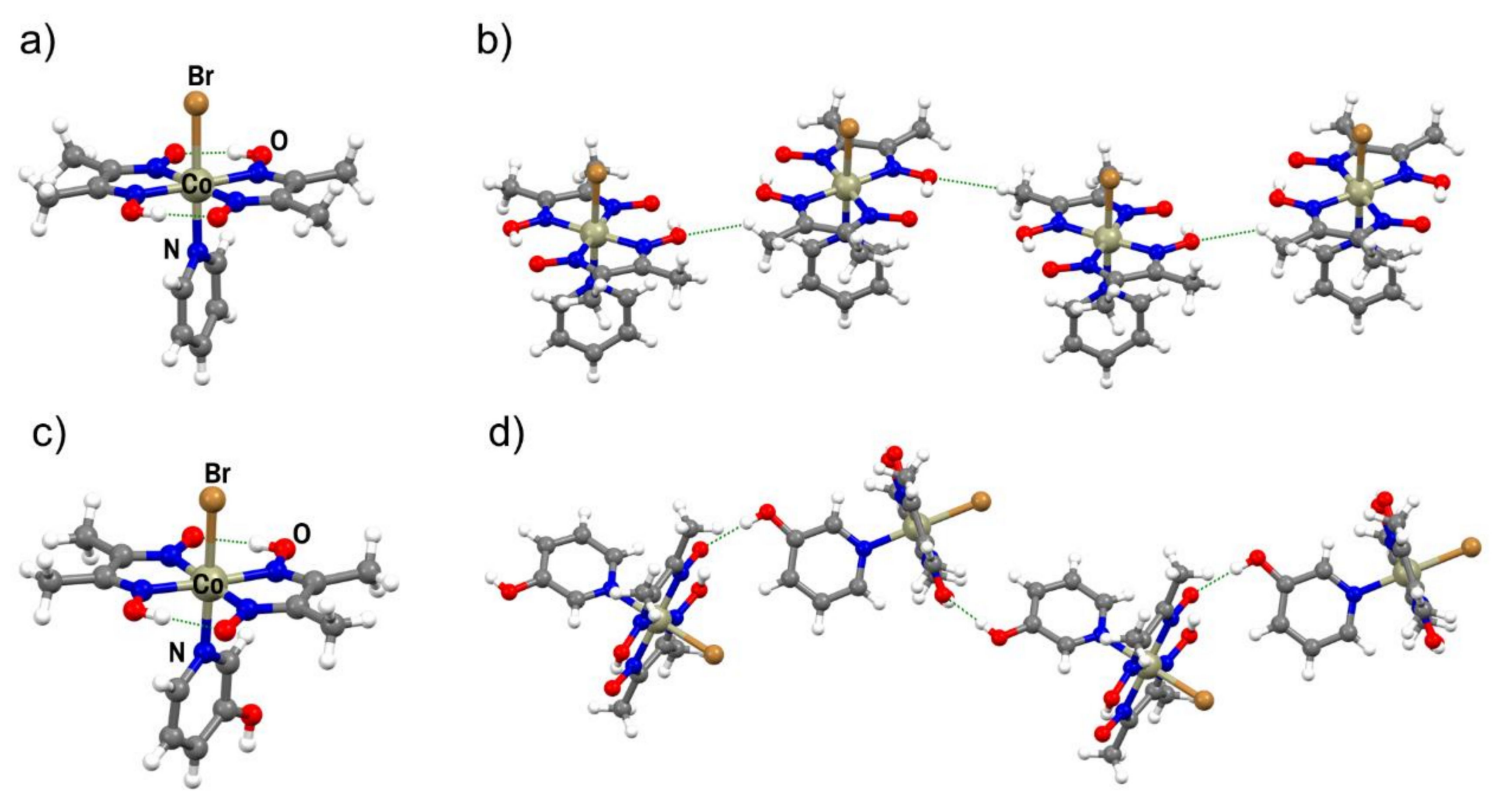
(**a**) Molecular structure of cobaloxime **I**; (**b**) C–H∙∙∙O contacts in the crystal structure of **I**; (**c**) the molecular structure of **II**; (**d**) O–H∙∙∙O hydrogen-bonded chain in the crystal structure of **II**.

Although all three metal complexes were successfully prepared as fine brown powders and characterized by XRPD and thermal analysis, molecular and crystal structures were successfully determined only for **I** and **II** (Figure 1a,c, Figures S1 and S2; Table S3). In both complexes, the metal cation is hexacoordinated, with donor atoms placed at the vertices of a distorted octahedron. The complexes are further stabilized by two intramolecular O–H∙∙∙O hydrogen bonds, which is a specific and well known feature of this class of compounds. In **II,** the molecules are connected by hydrogen bonding into chains by O–H∙∙∙O interactions, in which the hydroxyl group of 3-hydroxypyridine is the hydrogen bond donor and the oxime oxygen atom is the hydrogen bond acceptor (*d* = 2.715(5) Å, ∠(O–H∙∙∙O) = 144.3(5)°; Figure 1d). Chains are then connected in the 3D structure by C–H∙∙∙O and C–H∙∙∙π interactions. In the crystal structure of complex **I**, molecules are connected into chains by weak C_methyl_–H∙∙∙O contacts (*d* = 3.261(7) Å, ∠(C–H∙∙∙O) = 127.7(2)°) and further into the 3D structure by C–H∙∙∙O contacts, where C–H is part of the pyridine ring or methyl group of a **dmg** ligand (Figure 1b). This type of molecular interconnection is expected due to the absence of functional groups that could participate in stronger and more directional interactions.

The linear halogen bond donors (**14tfib** and **14tfbb**) preferentially form 2:1 cocrystals, while the ‘’bent’’ and sterically hindered **12tfib** forms cocrystals of 1:1 stoichiometry with the used metal–organic units. In 2:1 cocrystals, molecules are connected into discrete complexes, while chains have been found in the cocrystal with the 1:1 donor/acceptor ratio. Halogen bond parameters in crystal structures of the prepared cocrystals are shown in Table 1.

**Figure 2:**
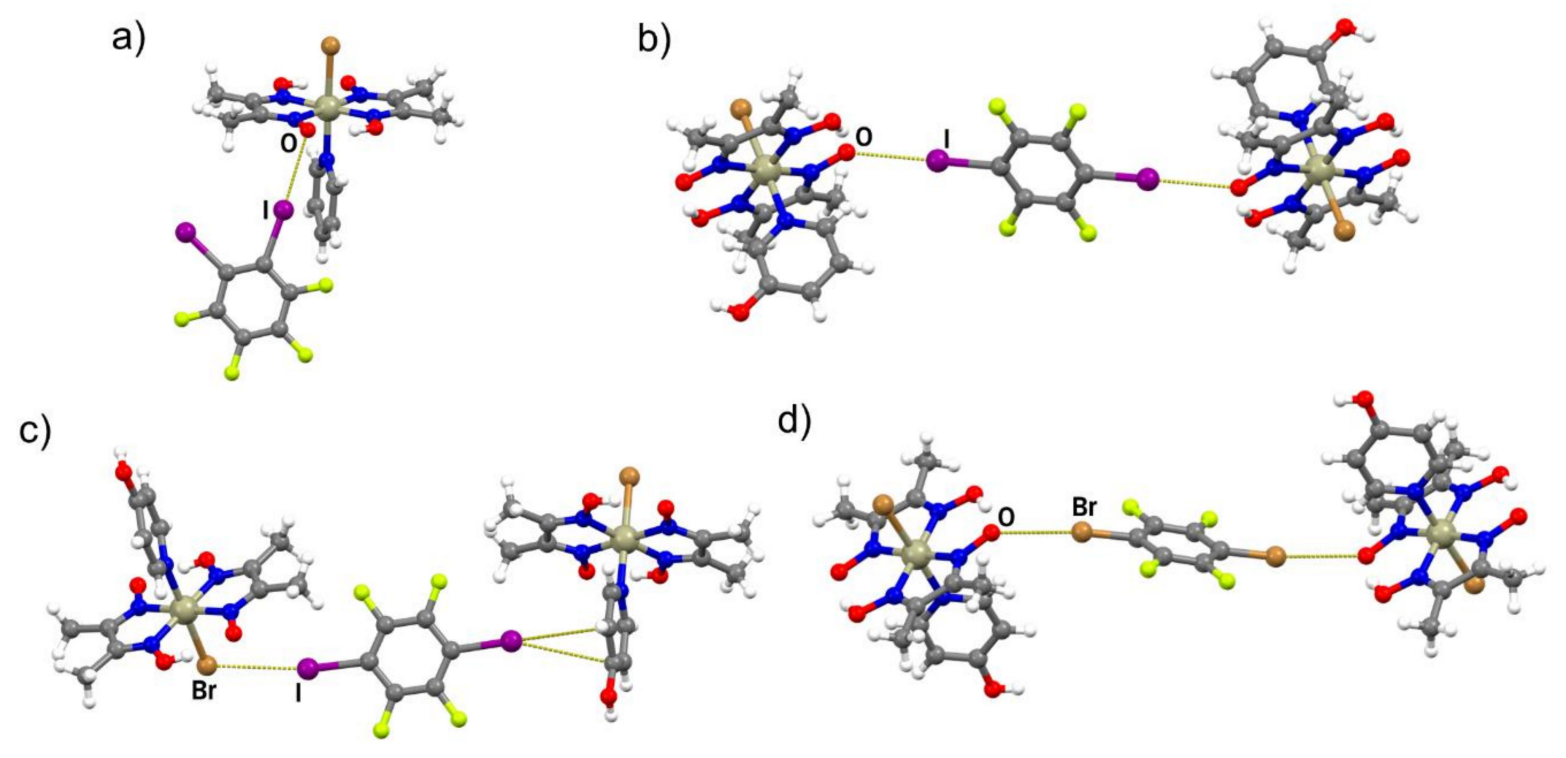
Discrete halogen-bonded units in (**a**) (**I**)(**12tfib**); (**b**) (**II**)_2_(**14tfib**); (**c**) (**III**)_2_(**14tfib**); (**d**) (**III)**_2_(**14tfbb**).

**Figure 3:**
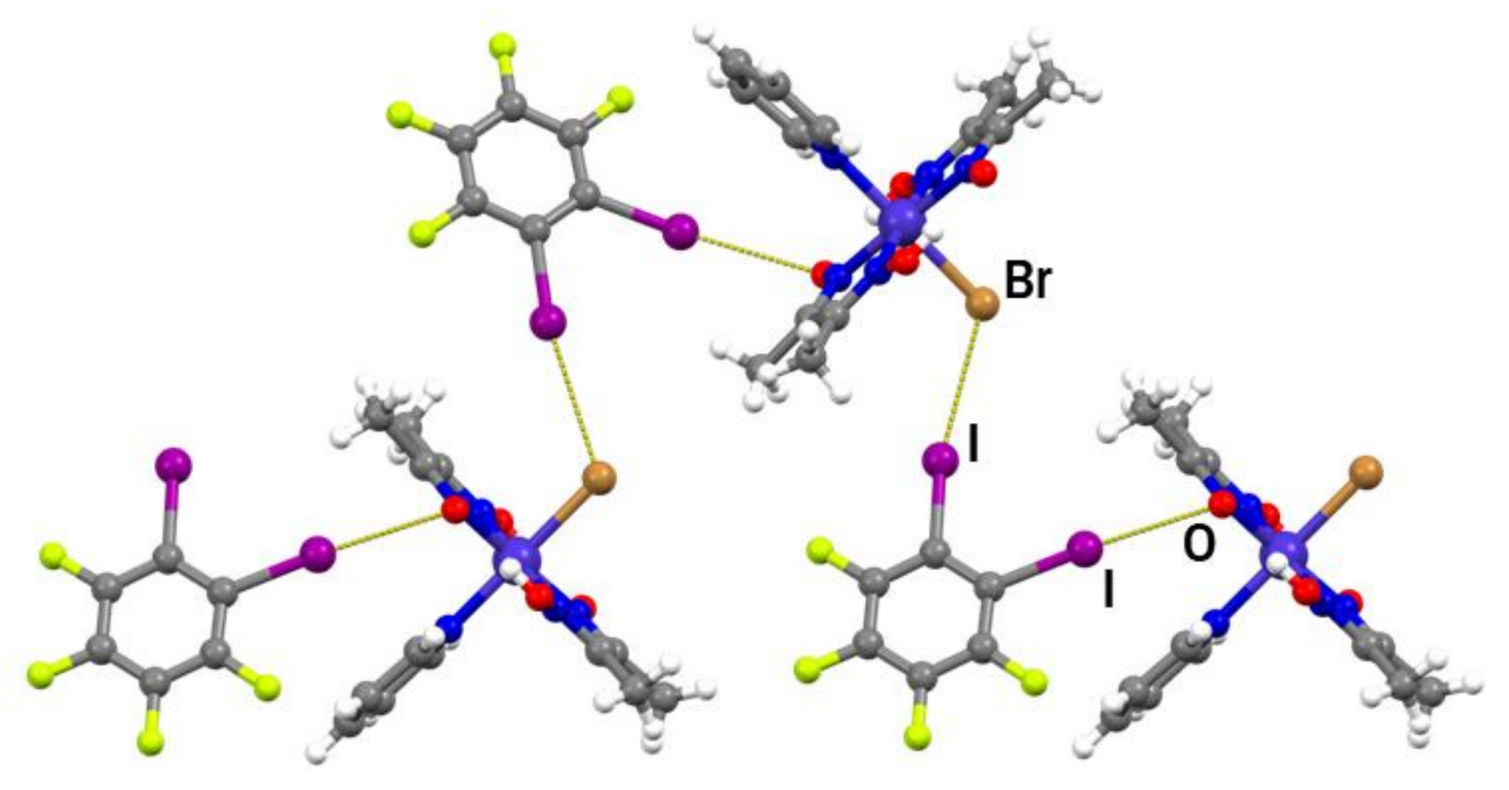
Halogen-bonded chain in the crystal structure of (**I**)(**12tfib**).

In the formula unit of (**I**)(**12tfib**), a halogen bond between one iodine atom and the deprotonated oxime oxygen was found (*r.s.(*I∙∙∙O) = 11.4%; Figure 2a and Figure S3). The second iodine atom of the **12tfib** molecule was found to form a somewhat longer halogen bond with the bromine atom coordinated to the metal center (*r.s.*(I∙∙∙Br) = 2.5 %). This interaction connects units of (**I**)(**12tfib**) into halogen-bonded zig-zag chains (Figure 3). Despite considerable differences in length, both the C–I∙∙∙O and the C–I∙∙∙Br halogen bond are roughly linear and have similar C–I∙∙∙Y angles. As expected, no other strong and directional noncovalent interactions have been observed in the crystal structure of (**I**)(**12tfib**), therefore chains are linked into 2D networks, and further into the 3D structure, by weak C–H∙∙∙F interactions.

**Figure 4:**
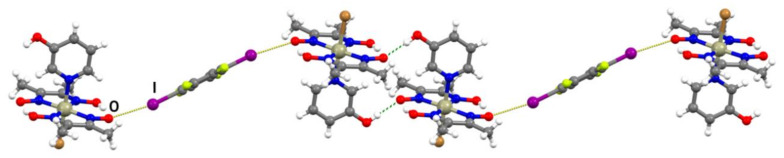
Halogen-bonded units of (**II**)_2_(**14tfib**) interconnected by O–H∙∙∙O hydrogen-bonded homosynthons into a chain.

Compound **II** forms cocrystals with two linear halogen bond donors: **14tfib** and **14tfbb**. The (**II**)_2_(**14tfib**) cocrystal consists of formula units formed by two symmetrically equivalent halogen bonds between iodine and oxime oxygen atoms (Figure 2b and Figure S4). The halogen bonds are relatively short (*r.s.*(I*∙∙∙*O) = 16.1%) and linear (∠(C–I*∙∙∙*O) = 169.2°), which is to be expected in cocrystals where **14tfib** is the halogen bond donor. The resulting halogen-bonded supramolecular complexes are connected into hydrogen-bonded chains by the metal–organic unit homosynthon $R_{2}^{2}\left(16\right)$, in which the 3-hydroxypyridine hydroxyl group is the hydrogen bond donor and the deprotonated oxime oxygen is the hydrogen bond acceptor (*d*(O*∙∙∙*O) = 2.667(7) Å; ∠(O–H∙∙∙O) = 165.3°) (Figure 4). Hydrogen-bonded chains are connected in the 3D structure by C–H∙∙∙F interactions. The crystal structure of (**II**)_2_(**14tfbb**) has not been determined in this study, but the evident similarity of (**II**)_2_(**14tfib**) and (**II**)_2_(**14tfbb**) powder patterns indicate that these two compounds are isostructural. Consequently, (**II**)_2_(**14tfbb**) also consists of halogen-bonded formula units of 2:1 halogen bond acceptor: donor stoichiometry, with two identical Br∙∙∙O halogen bonds.

**Figure 5:**
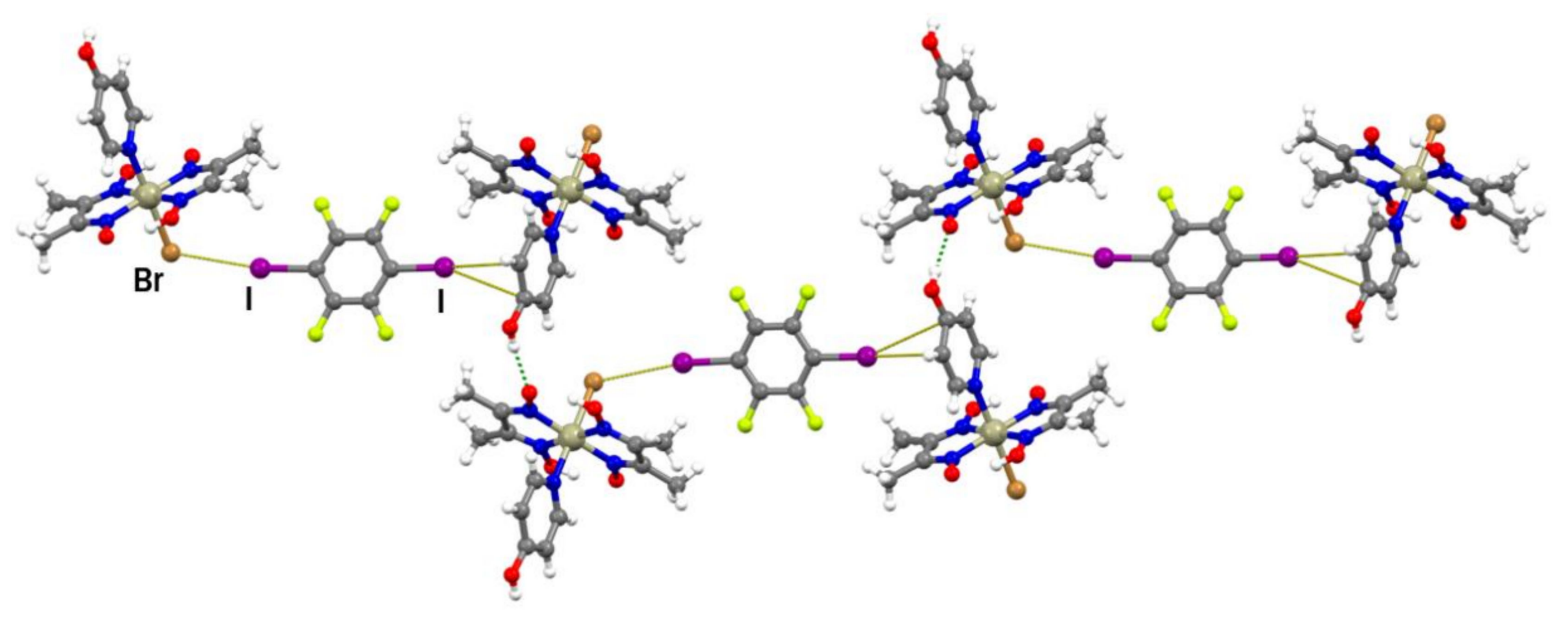
Halogen-bonded units of (**III**)_2_(**14tfib**) connected by O–H∙∙∙O hydrogen bonds into a chain.

Compound **III** also forms cocrystals with both linear halogen bond donors used in this work, but, unlike cocrystals of **II,** they are not isostructural. The (**III**)_2_(**14tfib**) cocrystal consists of metal–organic molecules bonded to **14tfib** by two nonequivalent C–I∙∙∙Br and C–I∙∙∙π halogen bonds (Figure 2c and Figure S5). Surprisingly, although complex **III** contains many other reliable binding sites, one of the available donor atoms participates in halogen bonding with the π-system of the 4-hydroxypyridine ring. This observation could be explained by the electron-donating nature of the hydroxyl group, which increases the electron density in the aromatic ring and enhances the halogen bond acceptor ability of the π−system. Nevertheless, the I∙∙∙π halogen bond is extremely long (*r.s.* = 0.7%) with a small C–I∙∙∙C(π) angle, indicating that it was mainly formed as a result of molecular close packing in the crystal (*C*_K_ = 66.2%). Halogen-bonded molecular complexes in (**III**)_2_(**14tfib**) are connected into chains by hydrogen bonding between 4-hydroxypyridine hydroxyl groups and deprotonated oxime oxygen atoms (*d*(O∙∙∙O) = 2.538(5) Å; ∠(O–H∙∙∙O) = 157.6°; Figure 5) and such chains are connected in the 3D structure by π-stacking and C–H∙∙∙F interactions.

**Figure 6:**
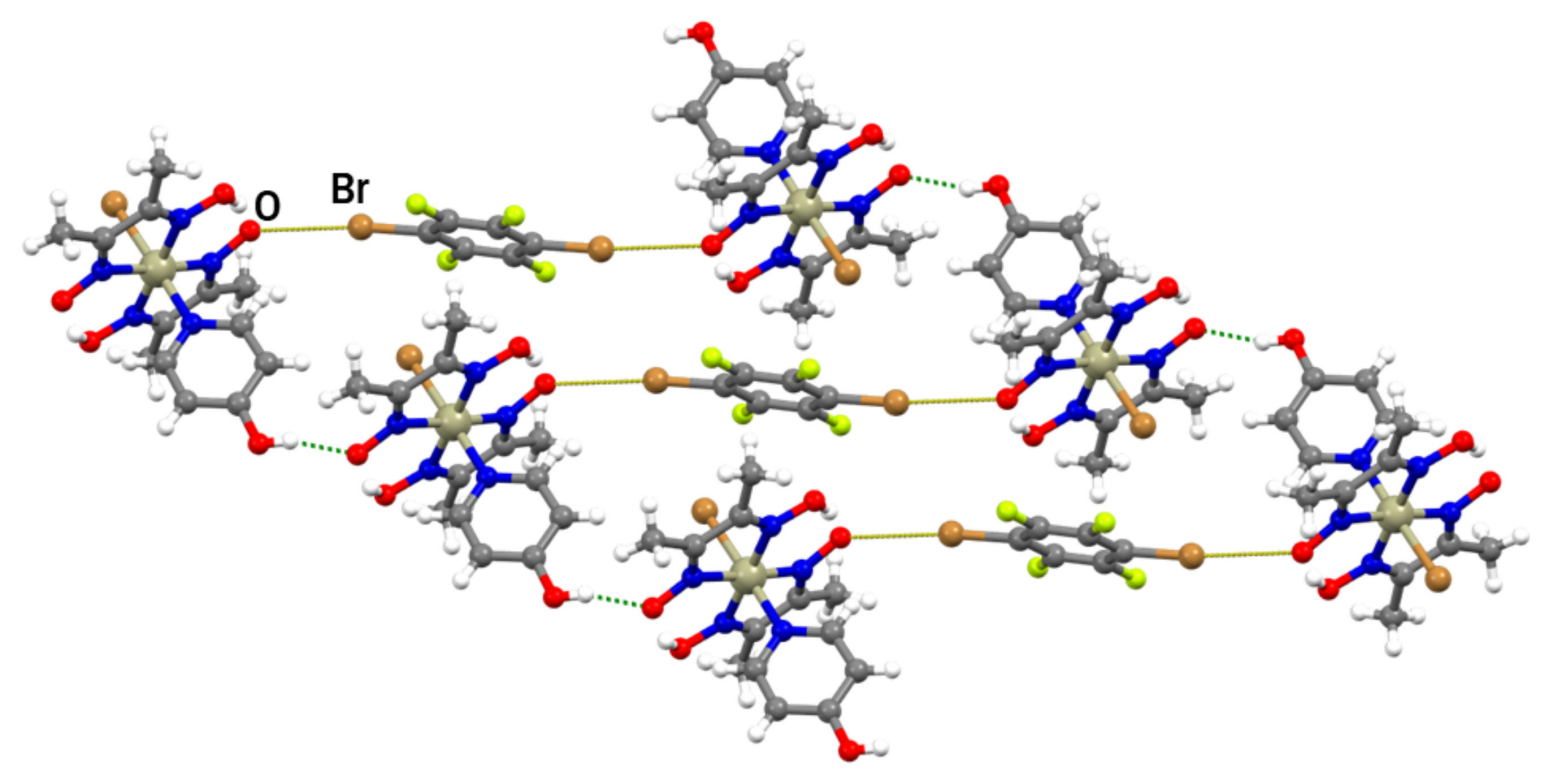
Halogen-bonded units of (**III**)_2_(**14tfbb**) connected by O–H∙∙∙O hydrogen-bonds into a chain.

In the (**III**)_2_(**14tfbb**) supramolecular complexes, the **14tfbb** molecule participates in two equivalent Br∙∙∙O halogen bonds in which the deprotonated oxime oxygen atom is the halogen bond acceptor (the same motif as the one found in (**II**)_2_(**14tfib**); Figure 2b and Figure S6). In comparison with (**II**)_2_(**14tfib**), the formed halogen bonds are slightly longer (*r.s*.(Br∙∙∙O) = 8.6%), but more linear (Table 1). The halogen-bonded complexes (**III**)_2_(**14tfbb**) are connected into chains by O–H∙∙∙O hydrogen bonds between 4-hydroxypyridine hydroxyl groups and the deprotonated oxime oxygen atom not involved in halogen bonding (*d*(O∙∙∙O) = 2.629(5) Å; ∠(O–H∙∙∙O) = 162.1°), with **14tfbb** molecules bridging between the chains (Figure 6). This structure is additionally stabilized by close contacts between the coordinated bromide of **III** with the electron-depleted carbon atoms (bonded to the bromine) of **14tfbb** molecules *d*(Br∙∙∙C) = 3.375(5) Å. The 3D structure of (**III**)_2_(**14tfbb**) consists of hydrogen-bonded chains interconnected by weak C–H∙∙∙O and C–H∙∙∙Br interactions.

### 3.3. Thermal Analysis

Upon heating, the obtained cocrystals decompose in two separate steps. The (**I**)(**12tfib**) cocrystal starts decomposing at the lowest temperature (122 °C) and is followed by (**II**)_2_(**14tfib**) and (**III**)_2_(**14tfib**), for which the decomposition start values are quite similar (184 °C and 188 °C, respectively). The highest decomposition temperature was measured for (**III**)_2_(**14tfbb**) (201 °C). Since this compound has the longest (weakest) halogen bond, its higher thermal stability could be ascribed to the higher value of the Kitaigorodskii crystal packing index (*C*_K_ = 68.3%). Furthermore, during the heating of (**II**)_2_(**14tfib**) and (**III**)_2_(**14tfib**), intense and broad exothermic signals at ≈270 °C were observed. Similar exothermic signals are also present in the DSC thermograms of the pure complexes **II** and **III,** but they are much narrower and more intense than for corresponding cocrystals (Figures S14–S25).

## 4. Conclusions

The cocrystals prepared and described herein indicate that cobaloximes could serve as reliable building blocks for metal–organic halogen-bonded cocrystals. Of all the acceptor π-sites present in the metal–organic molecules used, the oxime oxygen atom stood out as the best halogen bond acceptor, since it was involved in halogen bonding in a total of three cocrystals. The coordinated bromine atom is a slightly weaker acceptor (the I∙∙∙Br contact was found in two cocrystals), while the weakest one is the hydroxyl group of the coordinated pyridine molecule, which did not participate in halogen bonding with any of the used donors. In the (**III**)_2_(**14tfib**) cocrystal, binding of the π-system to the donor atom was observed, resulting in the formation of an extremely long and relatively weak halogen bond, which is presumably a consequence of close packing of halogen-bonded complexes in the crystal structure. In this study, a very small sample of cobaloxime complexes was used, but their simple synthesis and the large number of possible derivatives (with different halogenides or pseudohalogenides, as well as pyridine derivatives) could stimulate further research into halogen bonding in such systems. For this reason, we believe that this research has created new opportunities for the study of halogen-bonded cocrystals with metal–organic building blocks.

## Figures and Tables

**Table 1:** Halogen bond lengths, angles and relative shortening in crystal structures of the prepared cocrystals (X = I for **12tfib** and **14tfib;** X = Br for **14tfbb**). *r.s.* = 1−[*d*(X∙∙∙Y)/(*r*_vdW_,_X_ + *r*_vdW_,_Y_)].

Parameter	(I)(12tfib)	(II)_2_(14tfib)	(III)_2_(14tfib)	(III)_2_(14tfbb)
*d*(X∙∙∙O)/Å	3.126(7)	2.936(5)	**×**	3.079(4)
*r.s.*(X∙∙∙O)/%	10.7	16.1	**×**	8.6
∠(C–X∙∙∙O)/°	164.6(3)	169.2(2)	**×**	172.4(2)
*d*(X∙∙∙Br)/Å	3.443(2)	**×**	3.415(1)	**×**
*r.s.*(X∙∙∙Br)/%	10.1	**×**	10.8	**×**
∠(C–X∙∙∙Br)/°	166.4(3)	**×**	170.5(2)	**×**
*d*(X∙∙∙C(π)/Å	**×**	**×**	3.654(6)	**×**
*r.s.*(X∙∙∙C(π)/%	**×**	**×**	0.7	**×**
∠(C–X∙∙∙C(π)/°	**×**	**×**	157.8(2)	**×**

## Supplementary Materials

The following are available online at https://www.mdpi.com/1996-1944/13/10/2370/s1, Figure S1. Molecular structure of I showing the atom-labelling scheme. Displacement ellipsoids are drawn at the 50% probability level; Figure S2. Molecular structure of II showing the atom-labelling scheme. Displacement ellipsoids are drawn at the 50% probability level; Figure S3. Molecular structure of (I)(12tfib) showing the atom-labelling scheme. Displacement ellipsoids are drawn at the 50% probability level; Figure S4. Molecular structure of (II)_2_(14tfib) showing the atom-labelling scheme. Displacement ellipsoids are drawn at the 50% probability level; Figure S5. Molecular structure of (III)_2_(14tfib) showing the atom-labelling scheme. Displacement ellipsoids are drawn at the 50% probability level; Figure S6. Molecular structure of (III)_2_(14tfbb) showing the atom-labelling scheme. Displacement ellipsoids are drawn at the 50% probability level; Figure S7. Measured and calculated XRPD patterns of I; Figure S8. Measured and calculated XRPD patterns of (I)(12tfib); Figure S9. Measured and calculated XRPD patterns of II; Figure S10. Measured and calculated XRPD patterns of (II)_2_(14tfib); Figure S11. Measured XRPD patterns of (II)_2_(14tfbb) and (II)_2_(14tfib) and calculated XRPD pattern of (II)_2_(14tfib); Figure S12. Measured and calculated XRPD patterns of (III)_2_(14tfib); Figure S13. Measured and calculated XRPD patterns of (III)_2_(14tfbb); Figure S14. DSC thermogram of I; Figure S15. TG thermogram of I; Figure S16. DSC thermogram of (I)(12tfib); Figure S17. TG thermogram of (I)(12tfib); Figure S18. DSC thermogram of II; Figure S19. TG thermogram of II; Figure S20. DSC thermogram of (II)_2_(14tfib); Figure S21. TG thermogram of (II)_2_(14tfib); Figure S22. DSC thermogram of (III)_2_(14tfib); Figure S23. TG thermogram of (III)_2_(14tfib); Figure S24. DSC thermogram of (III)_2_(14tfbb); Figure S25. TG thermogram of (III)_2_(14tfbb); Table S1. Masses of the complexes (I, II, III) and donors (12tfib, 14tfib) used in the mechanochemical synthesis of cocrystals in 1:1 and 2:1 molar ratio. Experiments were performed by addition of 20 μL of nitromethane in reaction mixture. Table S2. Masses of the complexes (I, II, III) and donor 14tfbb used in the mechanochemical synthesis of cocrystals in 1:1 and 2:1 molar ratio. Experiments were performed by addition of 20 μL of nitromethane in reaction mixture. Table S3. An overview and crystallographic data of the prepared compounds. CCDC 1999040–1999045 contain crystallographic data for this paper. These data can be obtained free of charge from the Director, CCDC, 12 Union Road, Cambridge, CBZ 1EZ, UK (Fax: +44-1223-336033; email: deposit@ccdc.cam.ac.uk or www: http://www.ccdc.cam.ac.uk).
