# Supplementary material for: Cobaloximes as Building Blocks in Halogen-Bonded Cocrystals
Source: Materials (Basel). 2020 May 21;13(10):2370. doi: 10.3390/ma13102370 (PMC7287722; doi:10.3390/ma13102370)
Supplement: Supplementary file 1 [file materials-13-02370-s001.zip › materials_804374- supplement-final.docx/materials-804374-supplement-proof_corrected.docx]

Article

Cobaloximes as Building Blocks in Halogen-Bonded Cocrystals

Nikola Bedeković ^1^, Valentina Martinez ^1,2^, Edi Topić ^1^, Vladimir Stilinović ^1,^* and Dominik Cinčić ^1,^*

^1^ Department of Chemistry, Faculty of Science, University of Zagreb, Horvatovac 102A, 10 000 Zagreb, Croatia; [nbedekovic@chem.pmf.hr](mailto:nbedekovic@chem.pmf.hr) (N.B.); [vmartin@irb.hr](mailto:vmartin@irb.hr) (V.M.); [edi.topic@chem.pmf.hr](mailto:edi.topic@chem.pmf.hr) (E.T.)

^2^ Laboratory for Green Synthesis, Ruđer Bošković Institute, Bijenička cesta 54, 10 000 Zagreb, Croatia

***** Correspondence: vstilinovic@chem.pmf.hr (V.S.); [dominik@chem.pmf.hr](mailto:dominik@chem.pmf.hr) (D.C.); Tel.: +385 1 4606 371 (V.S.); Tel.: +385 1 4606 362 (D.C.)

Received: 29 April 2020; Accepted: 18 May 2020; Published: date

Table of Content

| Item | Page |
| --- | --- |
| Figures S1–S6 (ORTEP representations of the formula units of prepared compounds) | 1 |
| Figures S7–S13 (XRPD patterns of the prepared compounds) | 3 |
| Figures S14–S25 (DSC and TG curves of the prepared compounds) | 6 |
| Tables S1 & S2 (Experimental data for mechanochemical synthesis) | 12 |
| Crystallization experiment details | 12 |
| Table 3 (Crystallographic data) | 13 |
| Figures S26–S31 (Hirshfeld surfaces and fingerprint plots) | 15 |


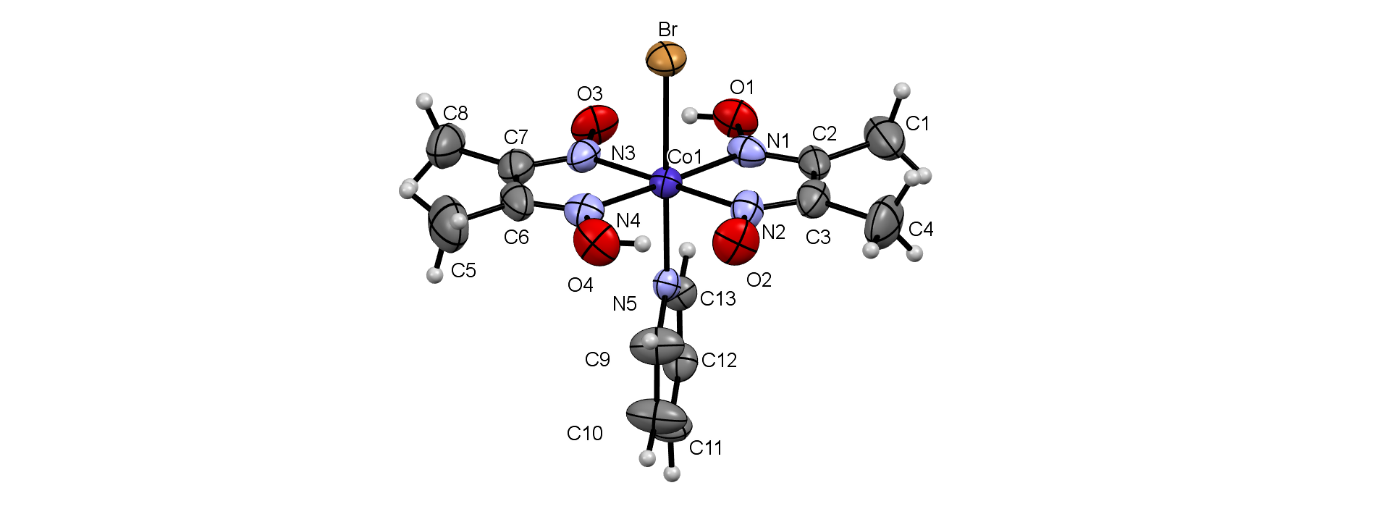


**Figure S1.** Molecular structure of **I** showing the atom-labelling scheme. Displacement ellipsoids are drawn at the 50% probability level.


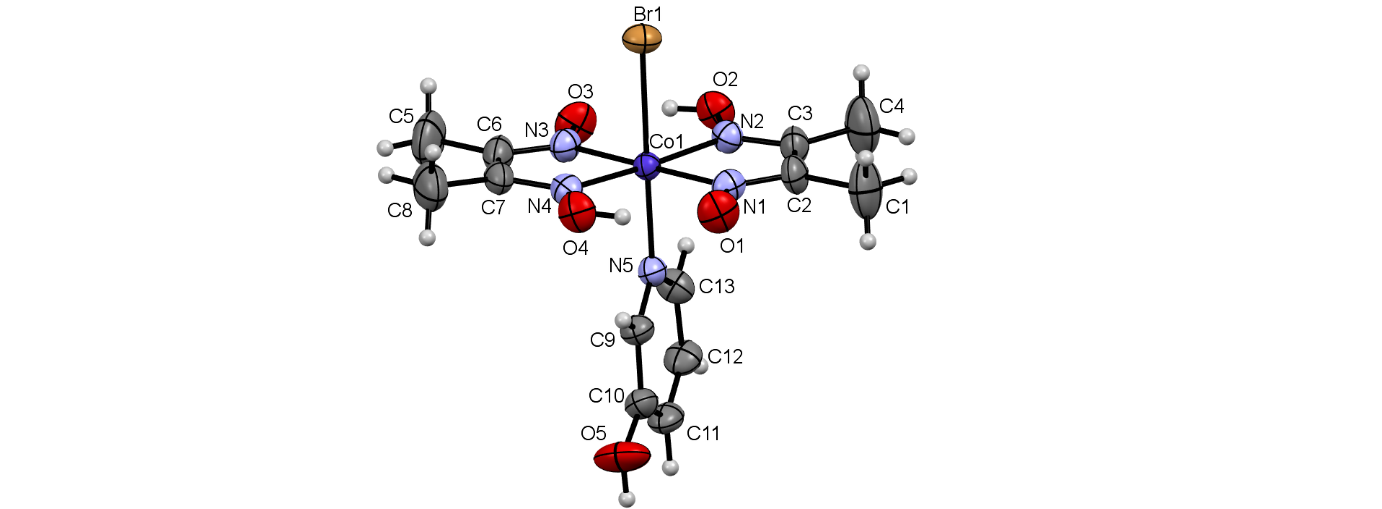


**Figure S2.** Molecular structure of **II** showing the atom-labelling scheme. Displacement ellipsoids are drawn at the 50 % probability level.


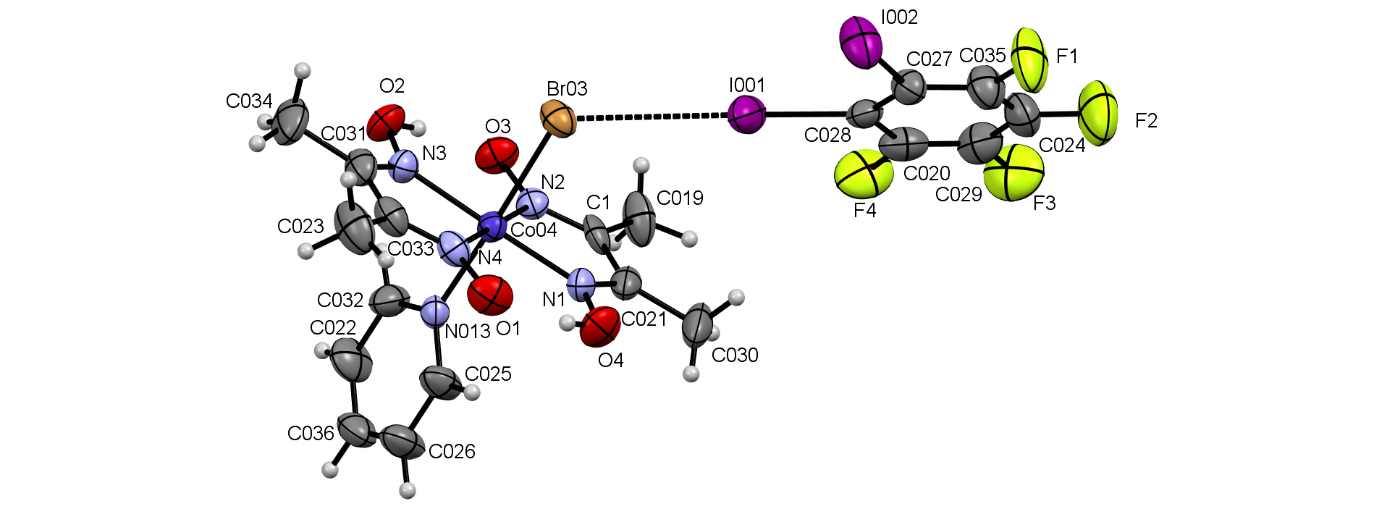


**Figure S3.** Molecular structure of (**I**)(**12tfib**) showing the atom-labelling scheme. Displacement ellipsoids are drawn at the 50% probability level.


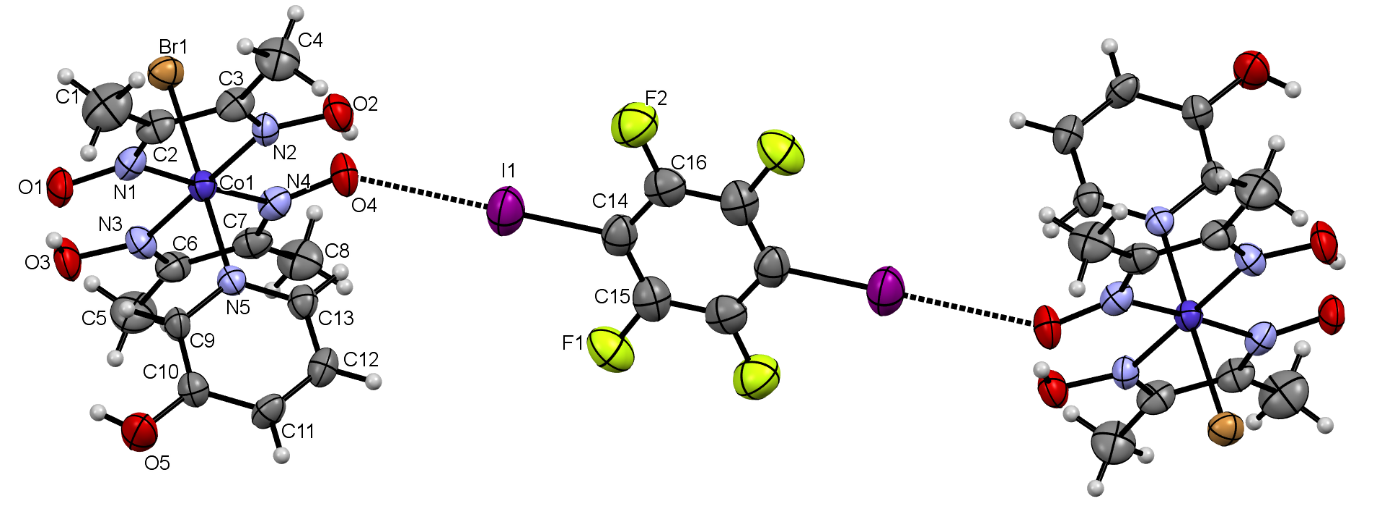


**Figure S4.** Molecular structure of (**II**)_2_(**14tfib**) showing the atom-labelling scheme. Displacement ellipsoids are drawn at the 50% probability level.


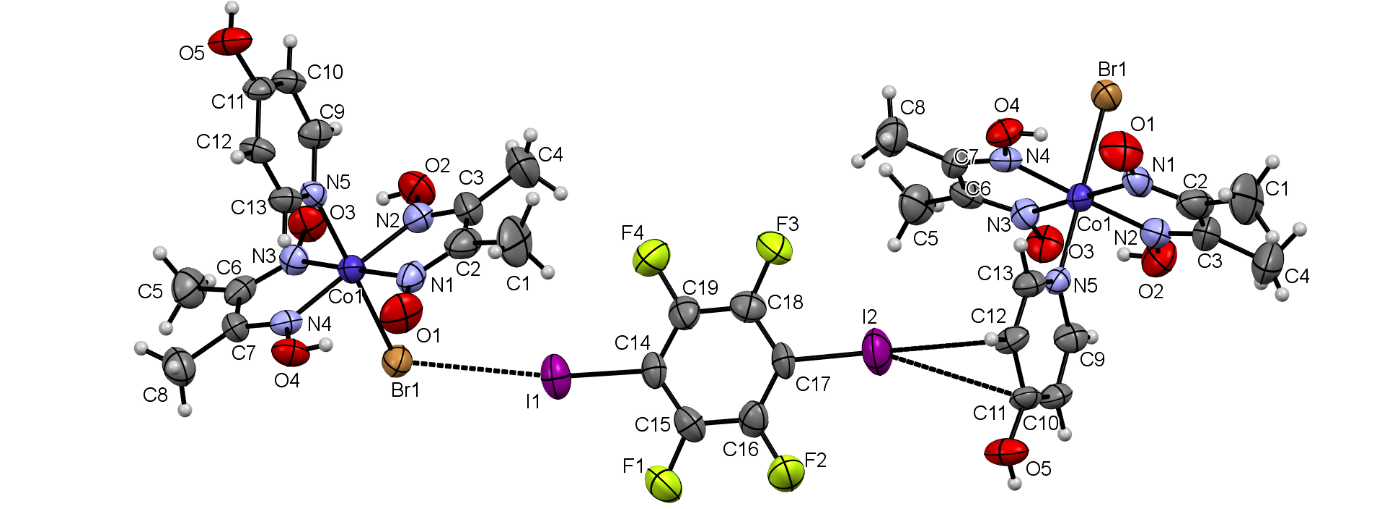


**Figure S5.** Molecular structure of (**III**)_2_(**14tfib**) showing the atom-labelling scheme. Displacement ellipsoids are drawn at the 50% probability level.


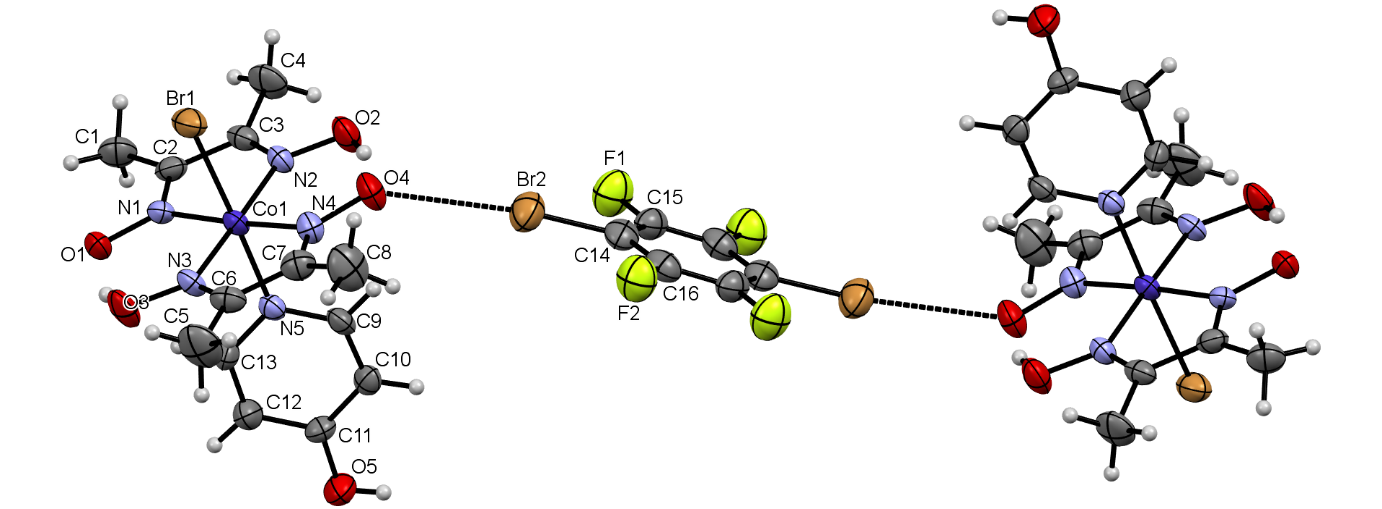


**Figure S6.** Molecular structure of (**III**)_2_(**14tfbb**) showing the atom-labelling scheme. Displacement ellipsoids are drawn at the 50% probability level.


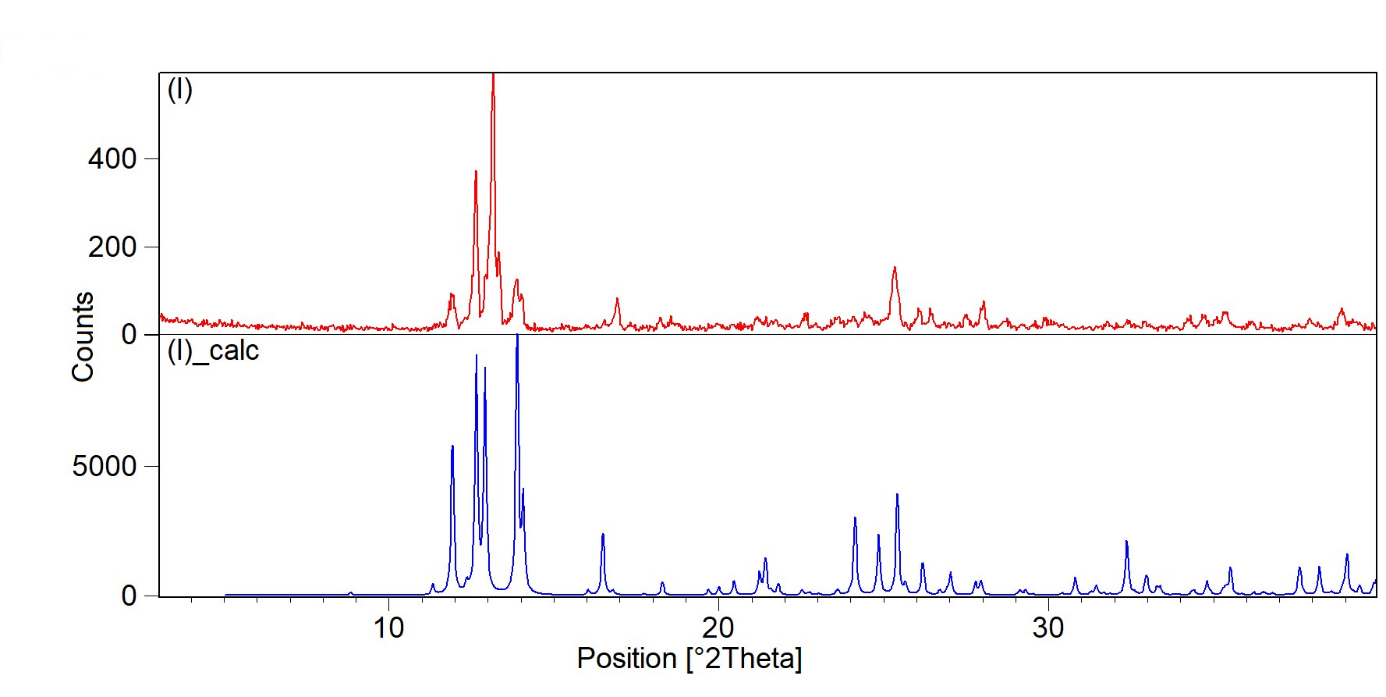


**Figure S7.** Measured and calculated XRPD patterns of **I**.


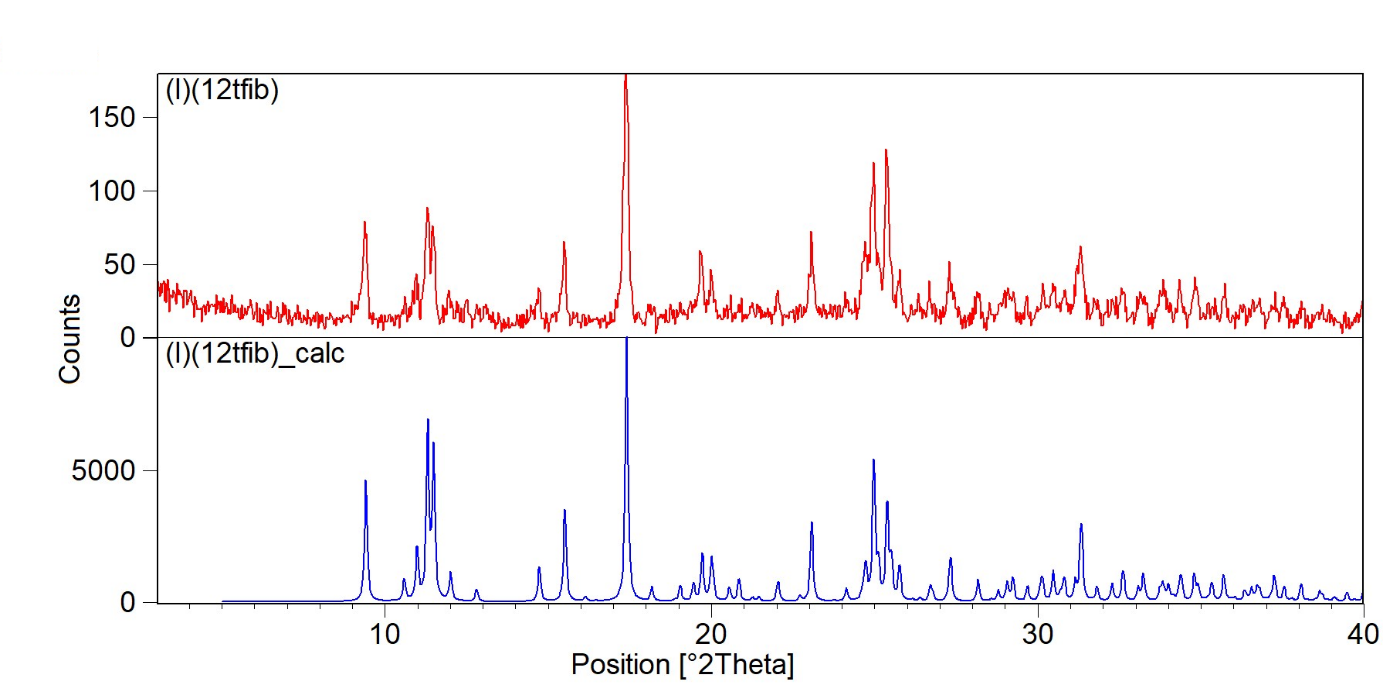


**Figure S8.** Measured and calculated XRPD patterns of (**I**)(**12tfib**).


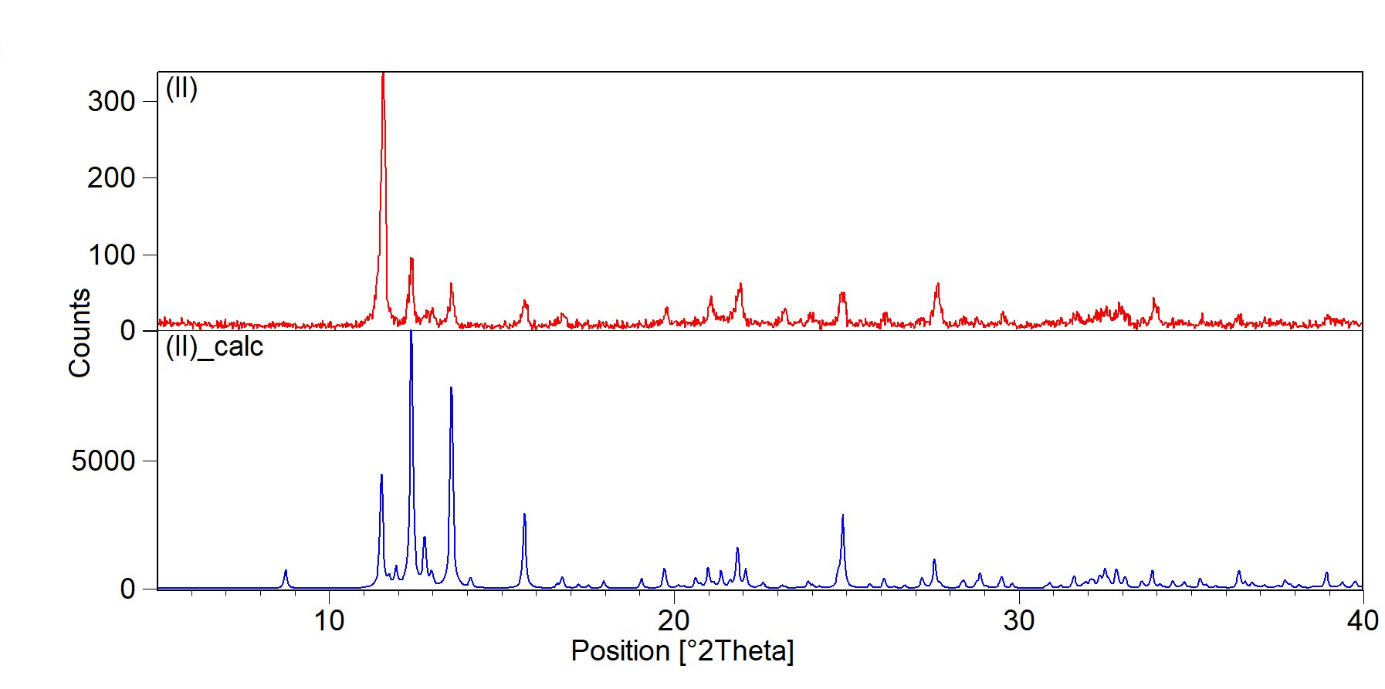


**Figure S9.** Measured and calculated XRPD patterns of **II**.


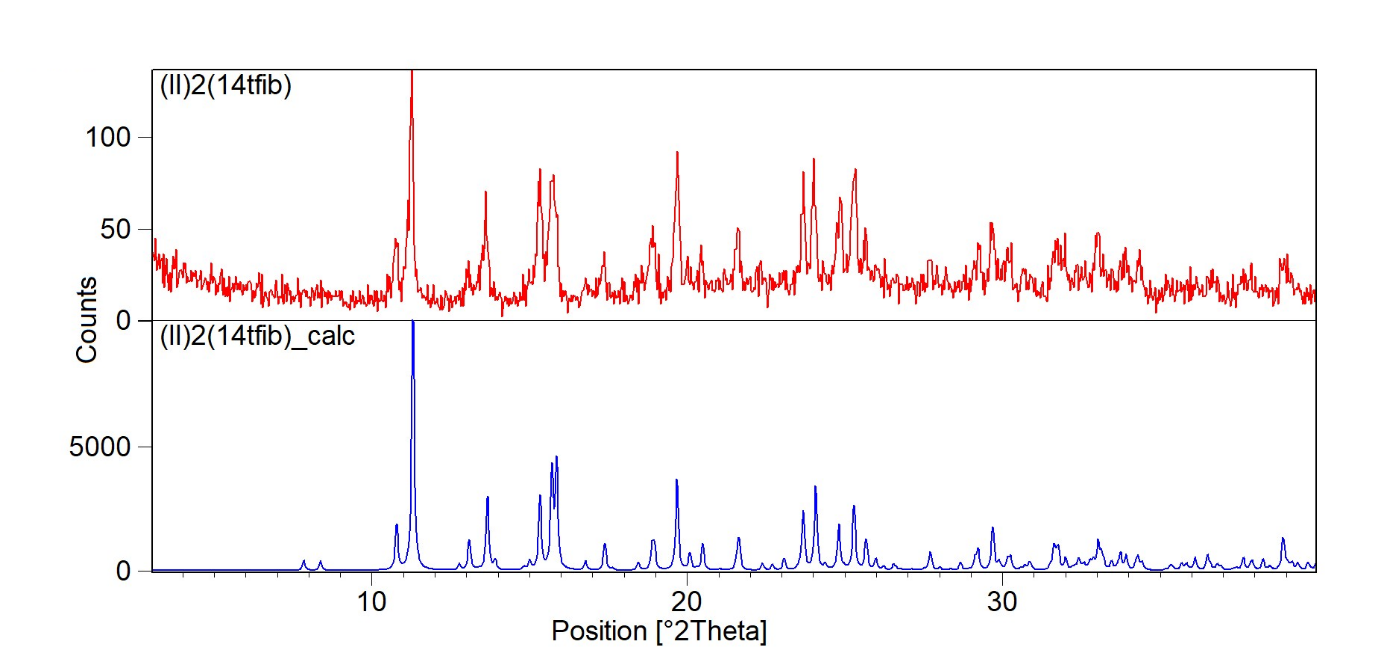


**Figure S10.** Measured and calculated XRPD patterns of(**II**)_2_(**14tfib**).


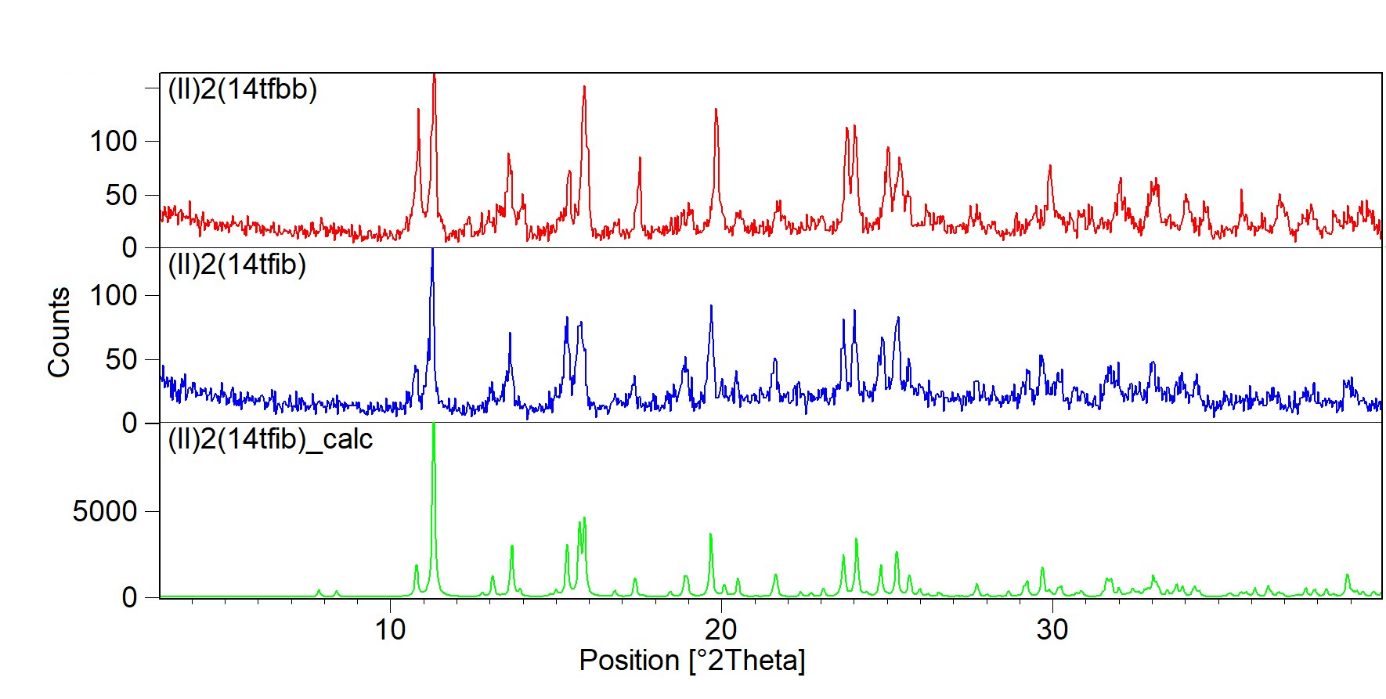


**Figure S11.** Measured XRPD patterns of (**II**)_2_(**14tfbb**) and (**II**)_2_(**14tfib**) and calculated XRPD pattern of (**II**)_2_(**14tfib**).


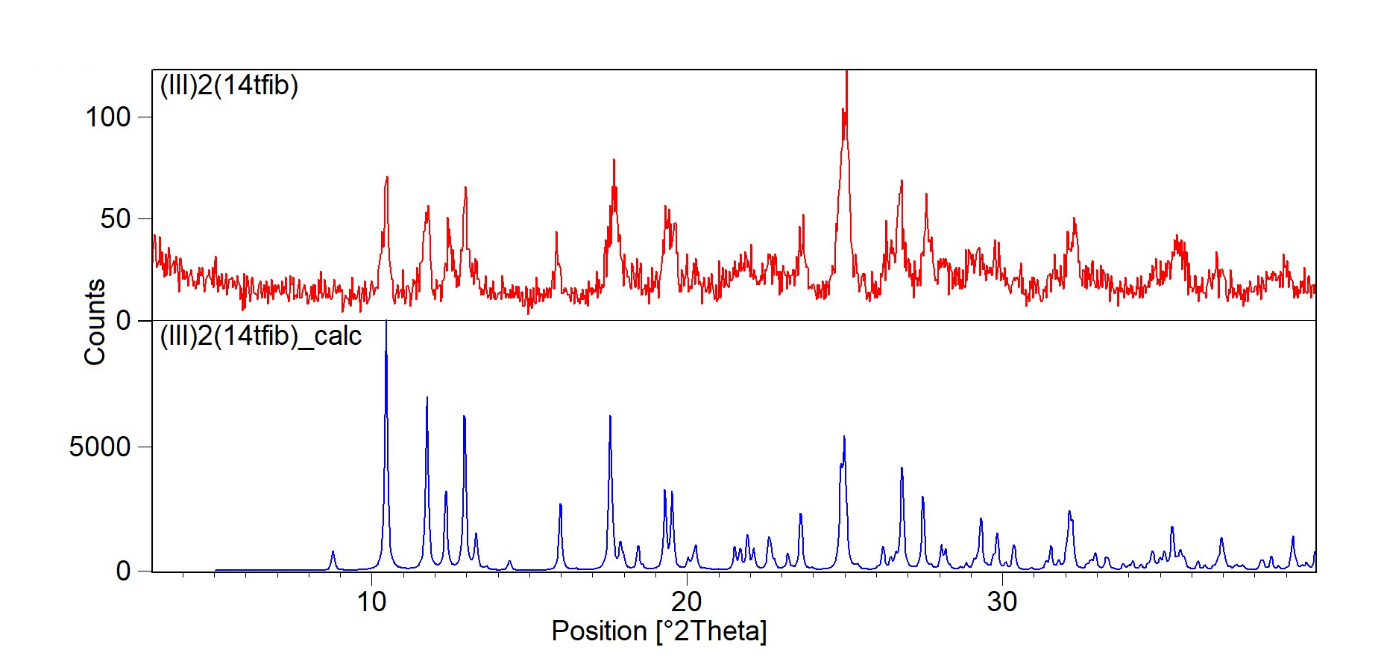


**Figure S12.** Measured and calculated XRPD patterns of(**III**)_2_(**14tfib**).


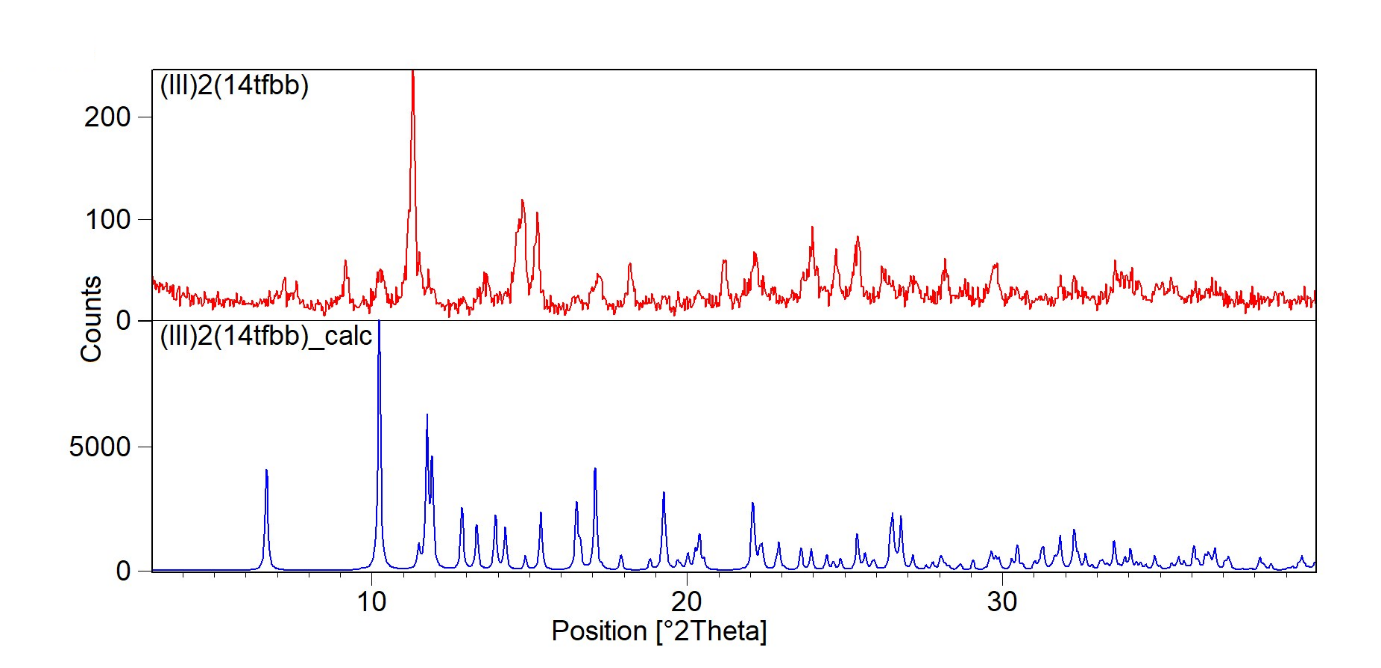


**Figure S13.** Measured and calculated XRPD patterns of(**III**)_2_(**14tfbb**).


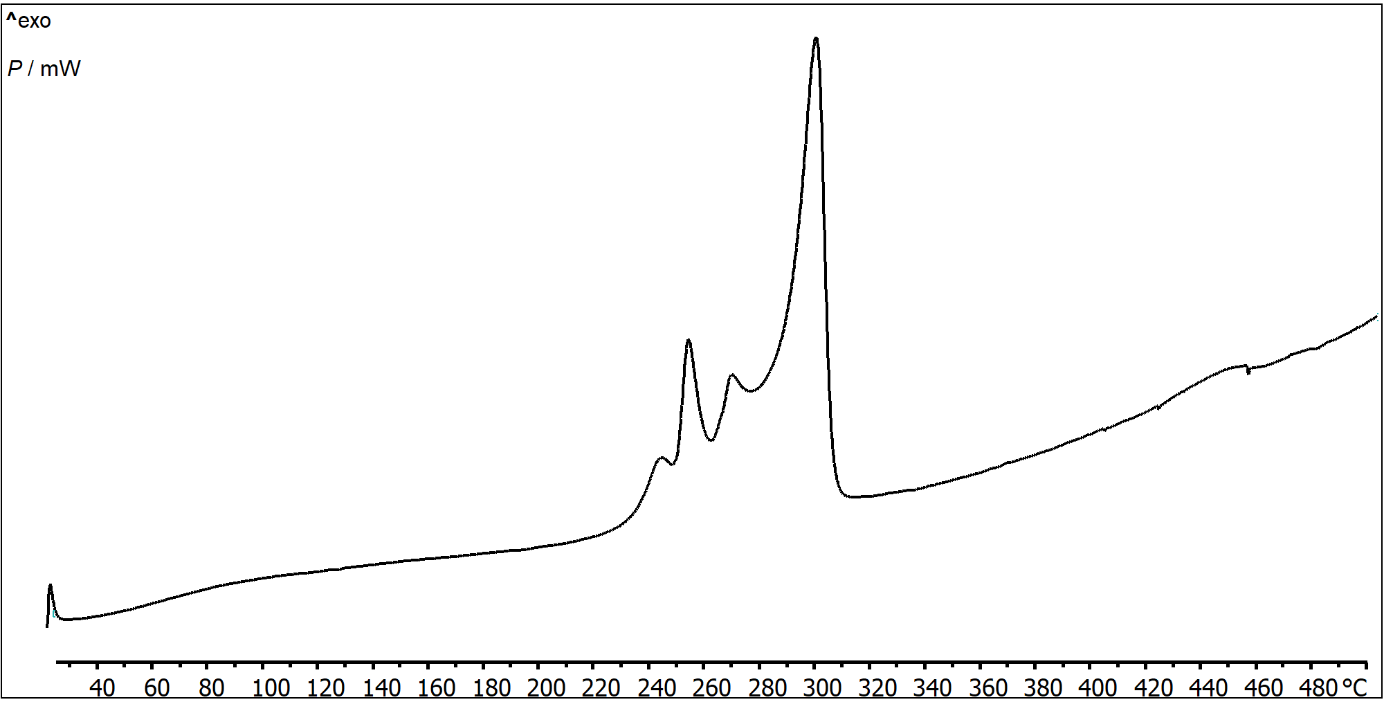


**Figure S14.** DSC thermogram of **I**.


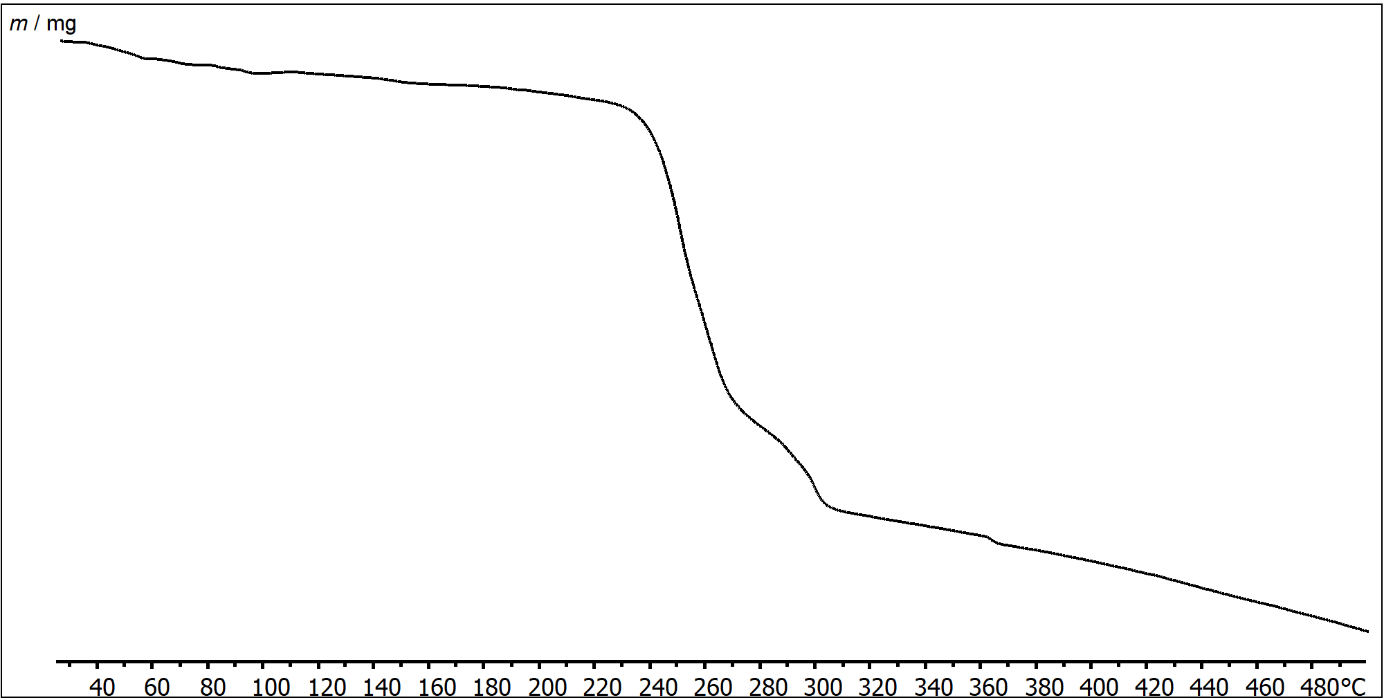


**Figure S15.** TG thermogram of **I**.


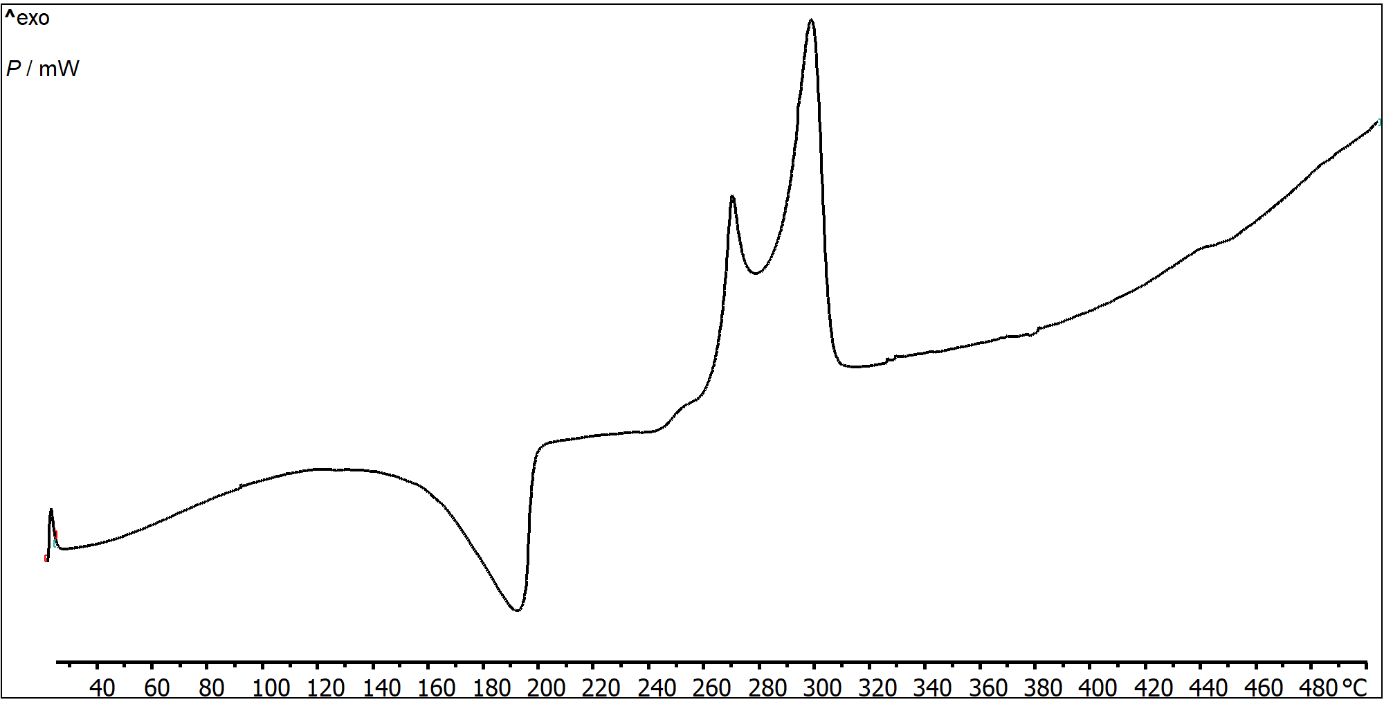


**Figure S16.** DSC thermogram of (**I**)(**12tfib**).


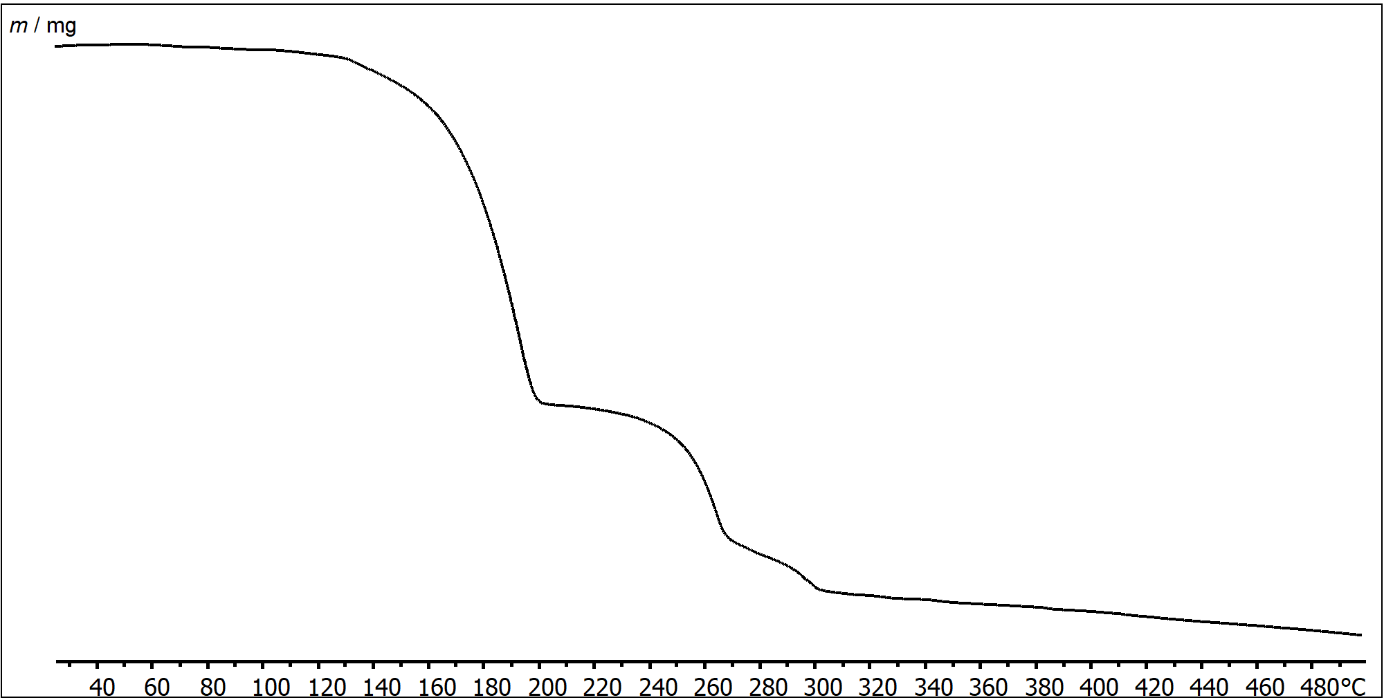


**Figure S17.** TG thermogram of (**I**)(**12tfib**).


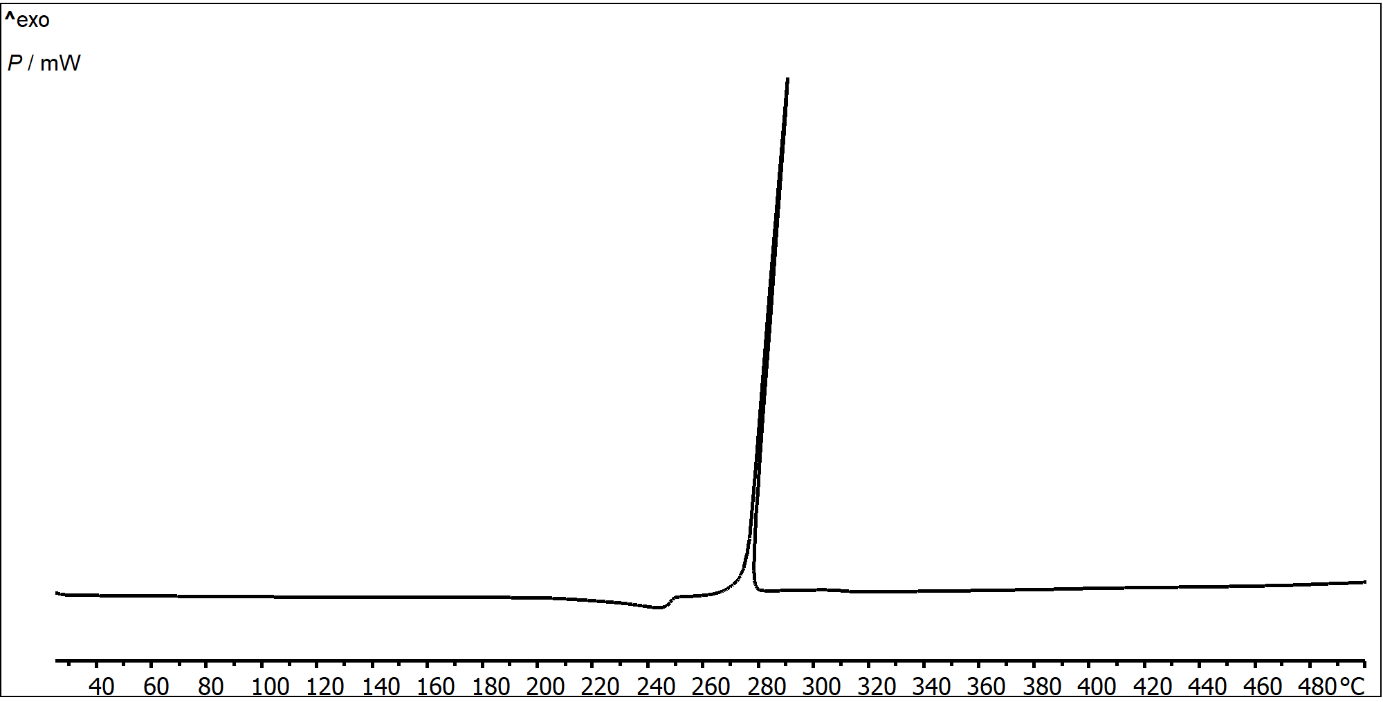


**Figure S18.** DSC thermogram of **II**.


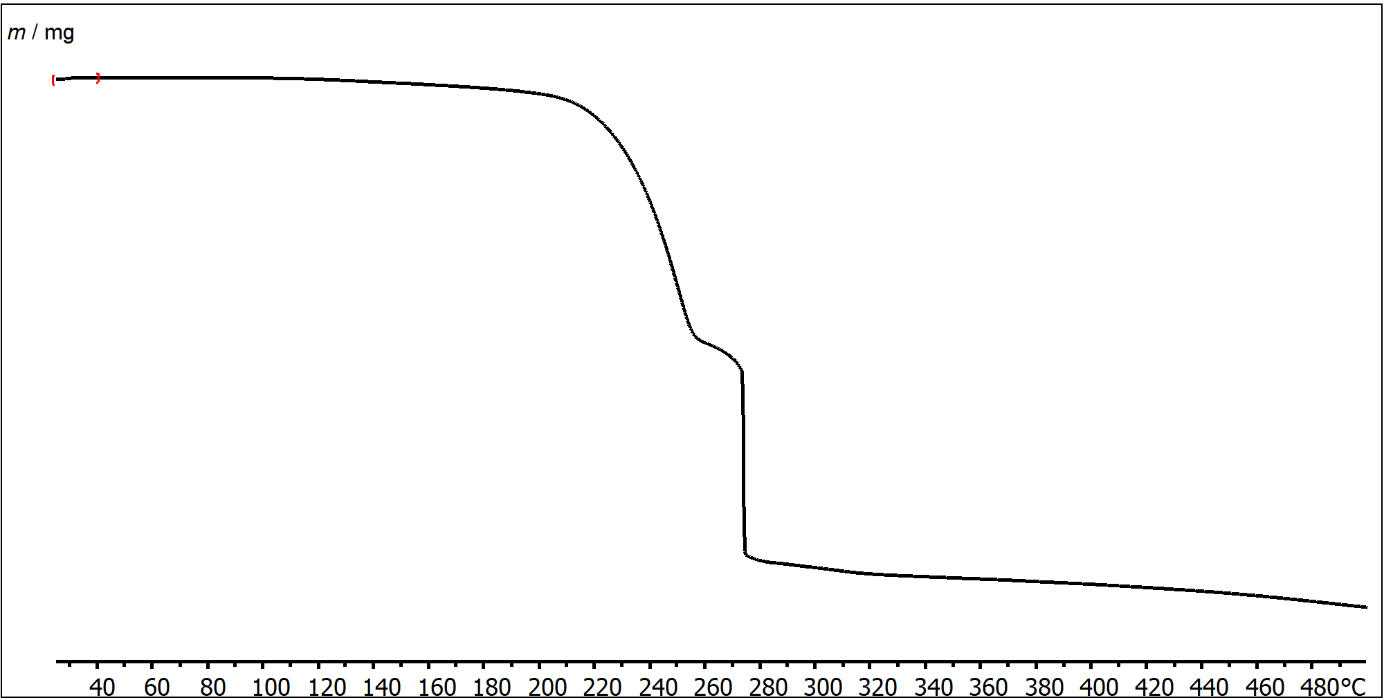


**Figure S19.** TG thermogram of **II**.


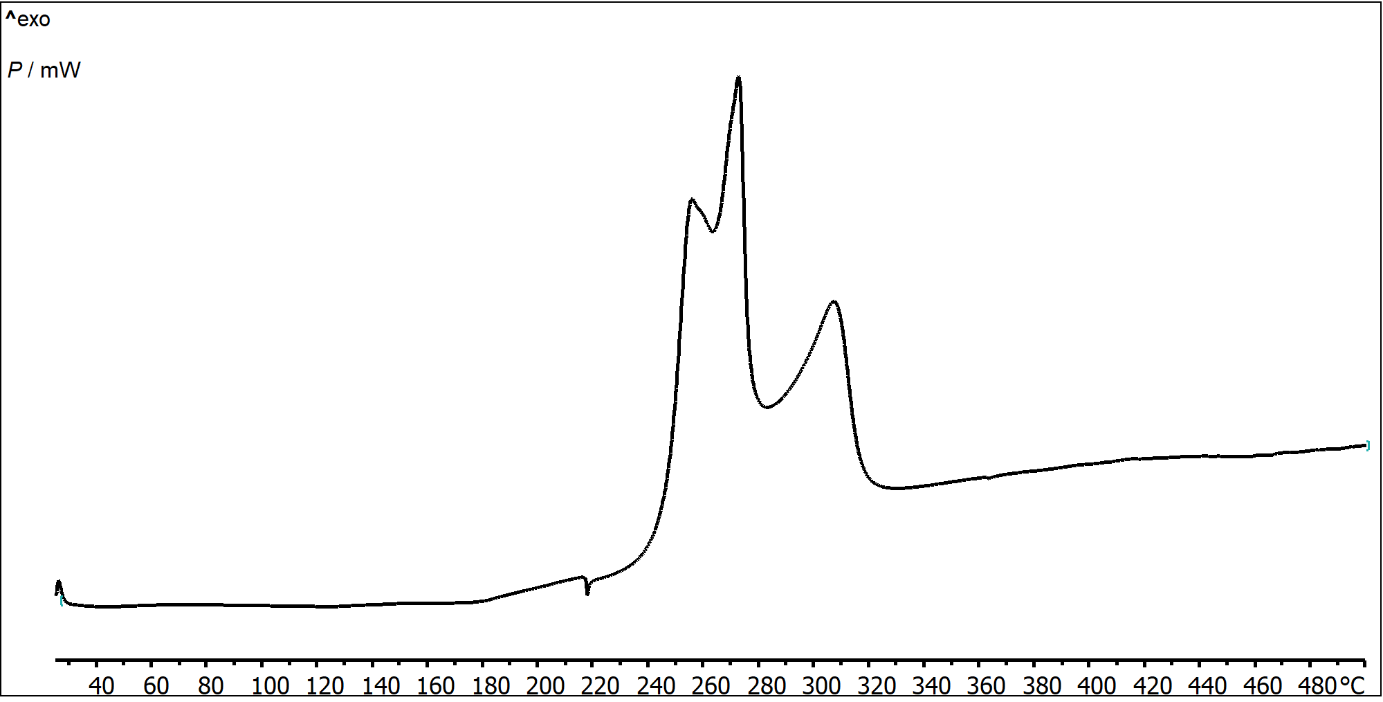


**Figure S20.** DSC thermogram of (**II**)_2_(**14tfib**).


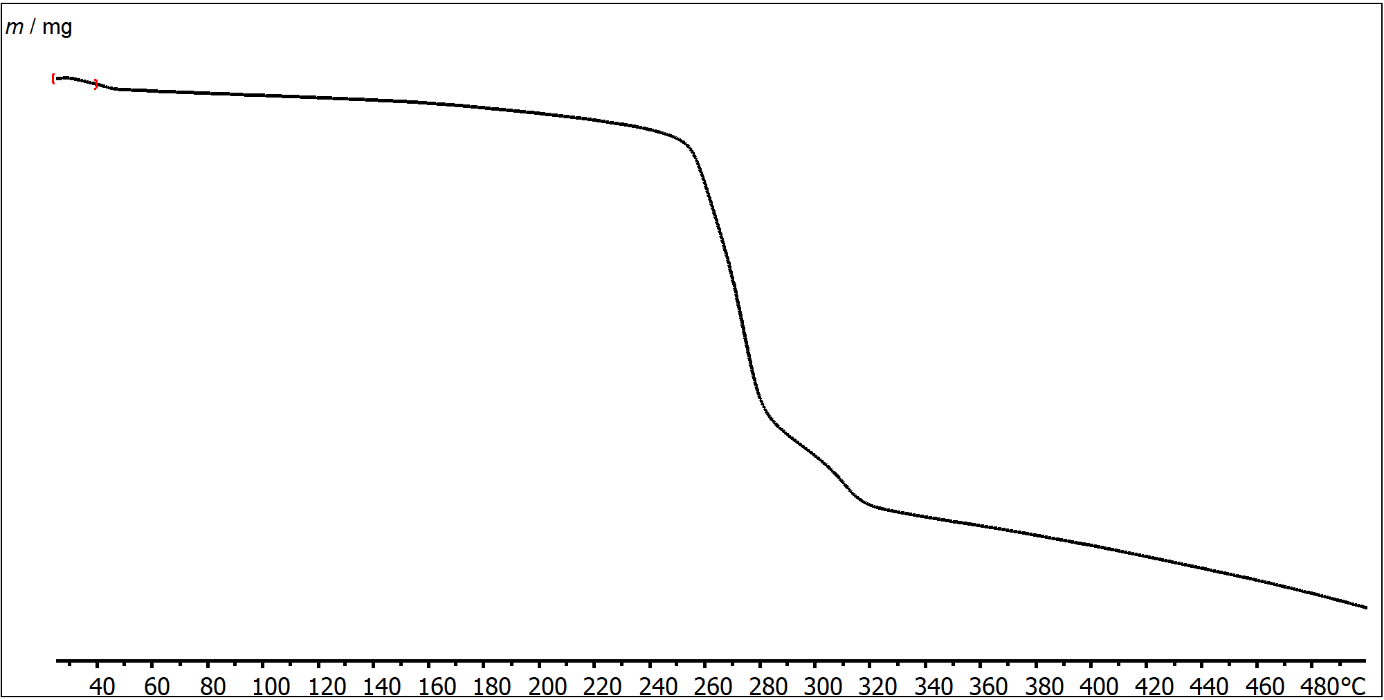


**Figure S21.** TG thermogram of (**II**)_2_(**14tfib**).


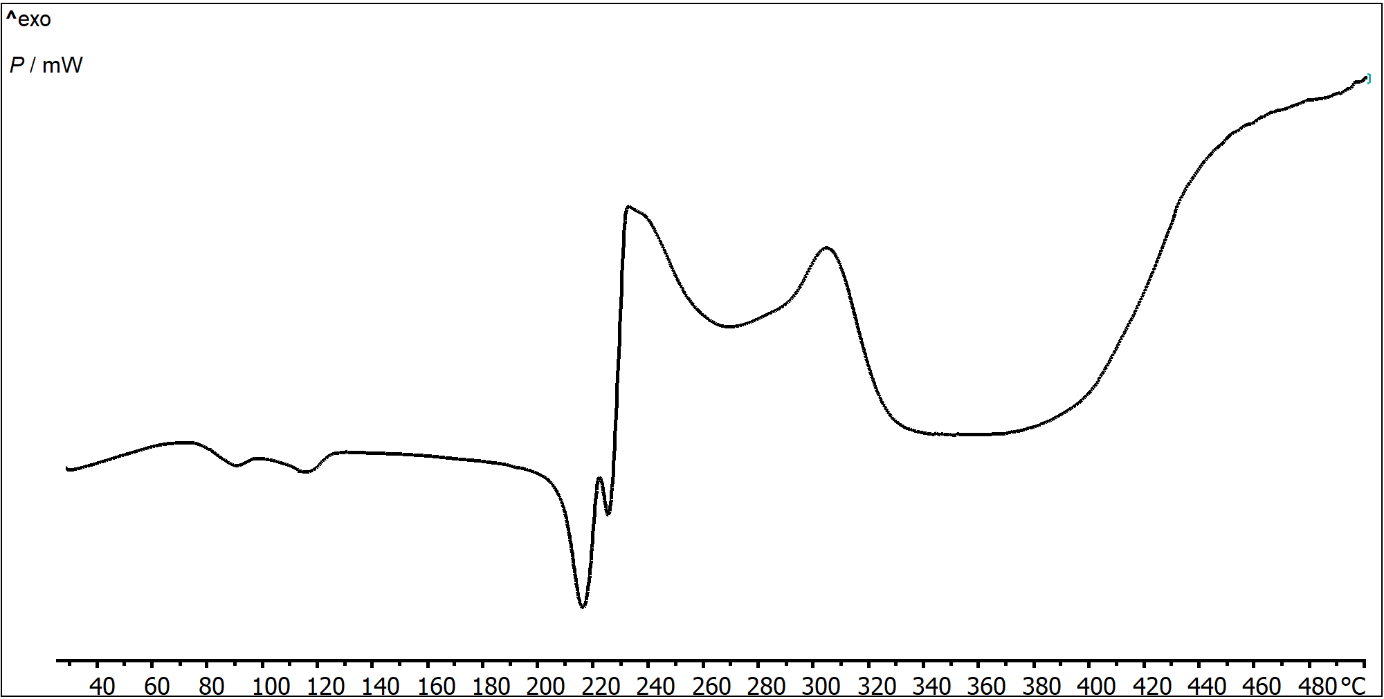


**Figure S22.** DSC thermogram of (**III**)_2_(**14tfib**).


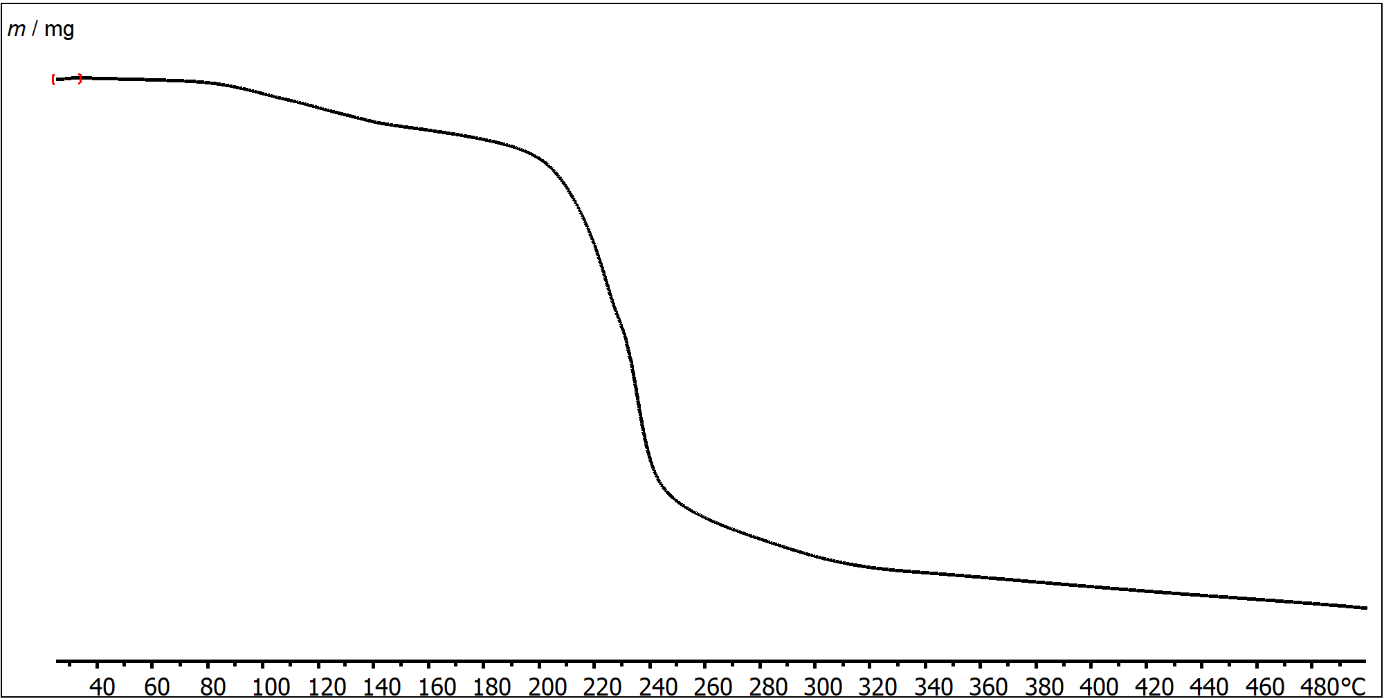


**Figure S23.** TG thermogram of (**III**)_2_(**14tfib**).


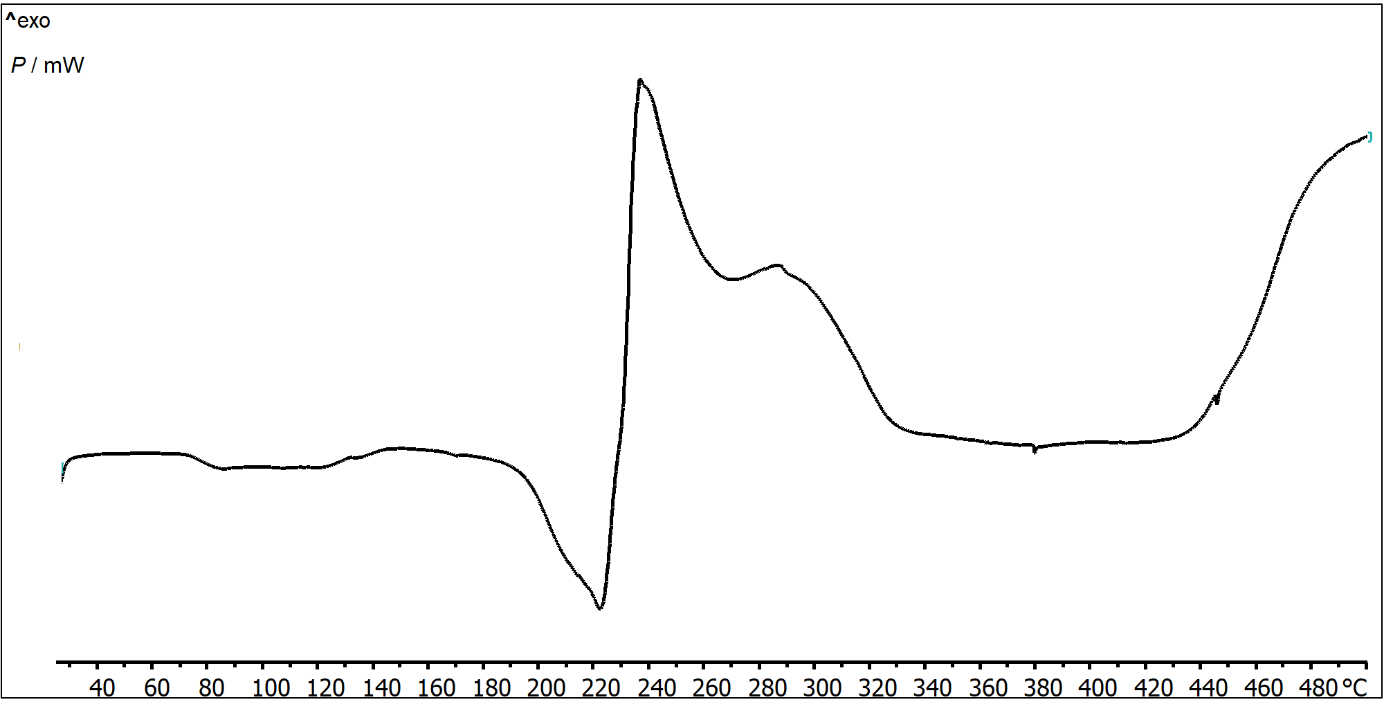


**Figure S24.** DSC thermogram of (**III**)_2_(**14tfbb**).


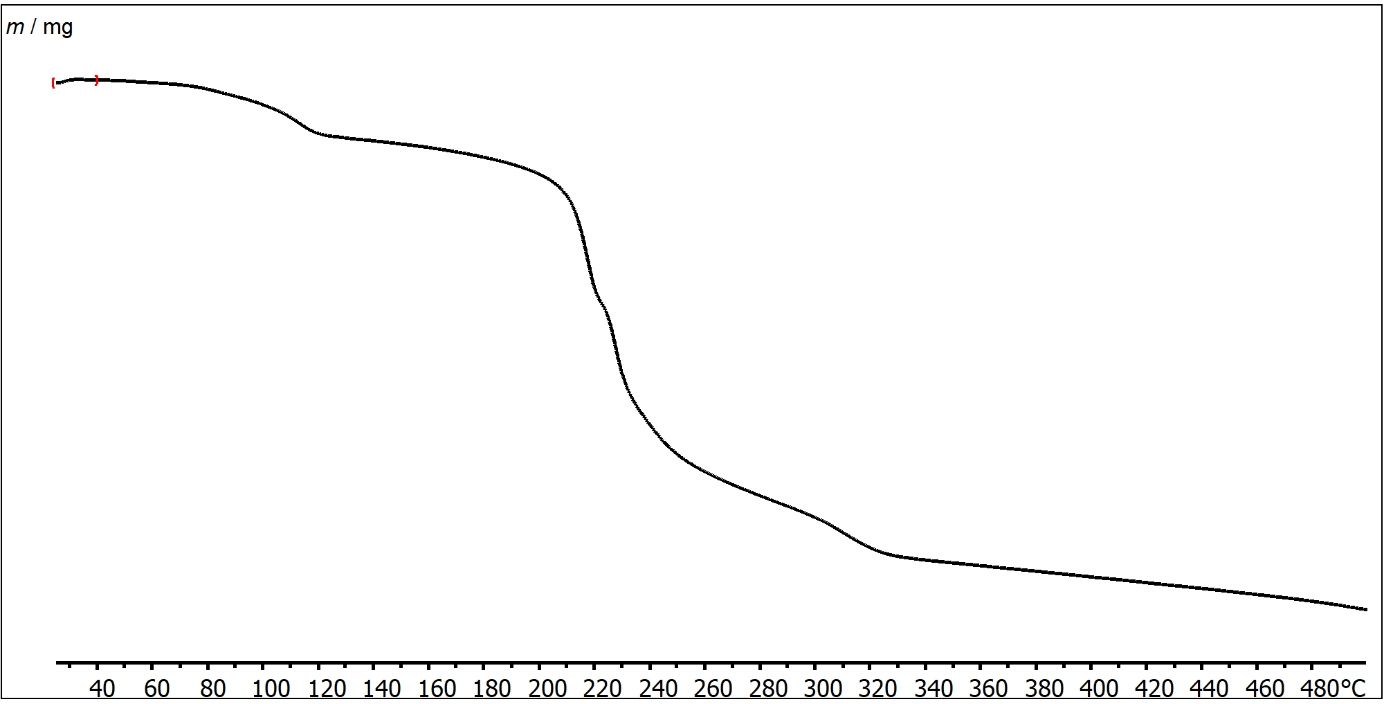


**Figure S25.** TG thermogram of (**III**)_2_(**14tfbb**).

**Table S1.** Masses of the complexes (**I**, **II**, **III**) and donors (**12tfib**, **14tfib**) used in the mechanochemical synthesis of cocrystals in 1:1 and 2:1 molar ratio. Experiments were performed by addition of 20 μL of nitromethane in reaction mixture.

| acceptor | **1:1** | | **2:1** | |
| --- | --- | --- | --- | --- |
|  | *m*(acceptor)/mg | *m*(donor)/mg | *m*(acceptor)/mg | *m*(donor)/mg |
| **I** | 42.1 | 37.9 | 55.2 | 24.8 |
| **II** | 42.8 | 37.2 | 55.8 | 24.2 |
| **III** | 42.8 | 37.2 | 55.8 | 24.2 |

**Table S2.** Masses of the complexes (**I**, **II**, **III**) and donor **14tfbb** used in the mechanochemical synthesis of cocrystals in 1:1 and 2:1 molar ratio. Experiments were performed by addition of 20 μL of nitromethane in reaction mixture.

| acceptor | **1:1** | | **2:1** | |
| --- | --- | --- | --- | --- |
|  | *m*(acceptor)/mg | *m*(donor)/mg | *m*(acceptor)/mg | *m*(donor)/mg |
| **I** | 47.3 | 32.7 | 59.5 | 20.5 |
| **II** | 48.0 | 32.0 | 60.0 | 20.0 |
| **III** | 48.0 | 32.0 | 60.0 | 20.0 |

Masses of reactants for crystallization experiments: Synthesis of (**I**)(**12tfib**), *m*(**I**) = 5.0 mg; *m*(**12tfib**) = 9.0 mg; Synthesis of (**II**)_2_(**14tfib**) and (**III**)_2_(**14tfib**), *m*(II) = 10.0 mg; *m*(**14tfib**) = 4.4 mg; Synthesis of (**III**)_2_(**14tfbb**), *m*(**III**) = 10,0; *m*(**14tfbb**) = 3,3 mg

**Table S3.** An overview and crystallographic data of the prepared compounds.

| **Compound** | **I** | **II** | **(I)(12tfib)** |
| --- | --- | --- | --- |
| Chemical formula | CoBrC_13_H_17_N_5_O_4_ | CoBrC_13_H_17_N_5_O_5_ | CoBrI_2_F_4_C_19_H_17_N_5_O_4_ |
| *M*/g mol^−1^ | 448.2 | 464.2 | 850.0 |
| Crystal system | monoclinic | monoclinic | monoclinic |
| Space group | *P*2_1_/*n* | *Pn* | *P*2_1_ |
| *a*/Å | 8.7426(6) | 8.7876(3) | 8.9048(5) |
| *b*/Å | 14.3293(12) | 14.8321(6) | 15.6550(5) |
| *c*/Å | 14.1011(9) | 13.9188(4) | 10.0166(5) |
| *α* | 90 | 90 | 90 |
| *β* | 96.923(6) | 94.610(3) | 110.215(6) |
| *γ* | 90 | 90 | 90 |
| *V*/Å^3^ | 1753.64(13) | 1808.29(5) | 1310.35(31) |
| *Z* | 4 | 4 | 2 |
| *T*/K | 295 | 295 | 295 |
| *ρ*_calc_/g cm^−3^ | 1.70 | 1.70 | 2.15 |
| *μ* (Mo-*K*_α_)/mm^−1^ | 3.284 | 3.192 | 4.599 |
| *θ*_min,max_ | 3.8  *θ*  27.0 | 3.8  *θ*  25.0 | 4.0  *θ*  25.0 |
| *h*_min,_ _max_ | −11  *h * 10 | −10  *h * 10 | −10  *h * 10 |
| *k*_min,_ _max_ | −16  *h * 18 | −17  *h *  | −18  *h * 18 |
| *l*_min,_ _max_ | −17  *h * 17 | −16  *h * 16 | −11  *h * 11 |
| *F*(000) | 904.0 | 936.0 | 808.0 |
| Number of measured data | 8420 | 24647 | 9044 |
| Number of unique data | 3776 | 6353 | 4575 |
| Number of observed data | 2007 | 5750 | 3622 |
| *R*_int_ | 0.054 | 0.023 | 0.035 |
| Number of refined parameters | 221 | 452 | 329 |
| *R*[*F*^2^ > 2*σF*^2^] | 0.049 | 0.021 | 0.034 |
| *wR*(*F*^2^) | 0.097 | 0.047 | 0.071 |
| *S* | 0.888 | 0.929 | 0.896 |
| Δ*ρ*_max_/e Å^−3^ | 0.616 | 0.271 | 1.022 |
| Δ*ρ*_min_/e Å^−3^ | −0.432 | −0.212 | −0.870 |

**Continuation of Table S3.**

| **Compound** | **(II)_2_(14tfib)** | **(III)_2_(14tfib)** | **(III)_2_(14tfbb)** |
| --- | --- | --- | --- |
| Chemical formula | Co_2_Br_2_I_2_F_4_C_32_H_34_N_10_O_10_ | Co_2_Br_2_I_2_F_4_C_32_H_34_N_10_O_10_ | Co_2_Br_4_F_4_C_32_H_34_N_10_O_10_ |
| *M*/g mol^−1^ | 1324.67 | 1330.27 | 1236.27 |
| Crystal system | monoclinic | monoclinic | orthorhombic |
| Space group | *P*2_1_/*n* | *P*2_1_/*n* | *Pbca* |
| *a*/Å | 8.3528(8) | 10.7618(9) | 8.9682(4) |
| *b*/Å | 22.5657(18) | 8.8634(6) | 18.2906(10) |
| *c*/Å | 11.9617(8) | 28.6282(57) | 26.5773(9) |
| *α* | 90 | 90 | 90 |
| *β* | 91.460(7) | 90.506(11) | 90 |
| *γ* | 90 | 90 | 90 |
| *V*/Å^3^ | 2253.89(5) | 2730.63(7) | 4359.57(3) |
| *Z* | 4 | 4 | 4 |
| *T*/K | 295 | 295 | 295 |
| *ρ*_calc_/g cm^−3^ | 1.96 | 2.11 | 1.88 |
| *μ* (Mo-*K*_α_)/mm^−1^ | 3.957 | 4.419 | 4.509 |
| *θ*_min,max_ | 4.7  *θ*  25.0 | 3.7  *θ*  25.0 | 3.8  *θ*  27.0 |
| *h*_min,_ _max_ | −9  *h * 9 | −12  *h * 12 | −11  *h * 8 |
| *k*_min,_ _max_ | −26  *h * 26 | −10  *h *  | −23  *h * 19 |
| *l*_min,_ _max_ | −14  *h * 14 | −34  *h * 34 | −33  *h * 33 |
| *F*(000) | 1292.0 | 1648.0 | 2440.0 |
| Number of measured data | 12857 | 16190 | 14040 |
| Number of unique data | 3937 | 4517 | 44587 |
| Number of observed data | 2561 | 2294 | 2478 |
| *R*_int_ | 0.074 | 0.063 | 0.065 |
| Number of refined parameters | 280 | 338 | 284 |
| *R*[*F*^2^ > 2*σF*^2^] | 0.051 | 0.035 | 0.054 |
| *wR*(*F*^2^) | 0.105 | 0.058 | 0.095 |
| *S* | 0.969 | 0.722 | 0.904 |
| Δ*ρ*_max_/e Å^−3^ | 0.896 | 0.509 | 0.588 |
| Δ*ρ*_min_/e Å^−3^ | −0.979 | −0.481 | −0.624 |


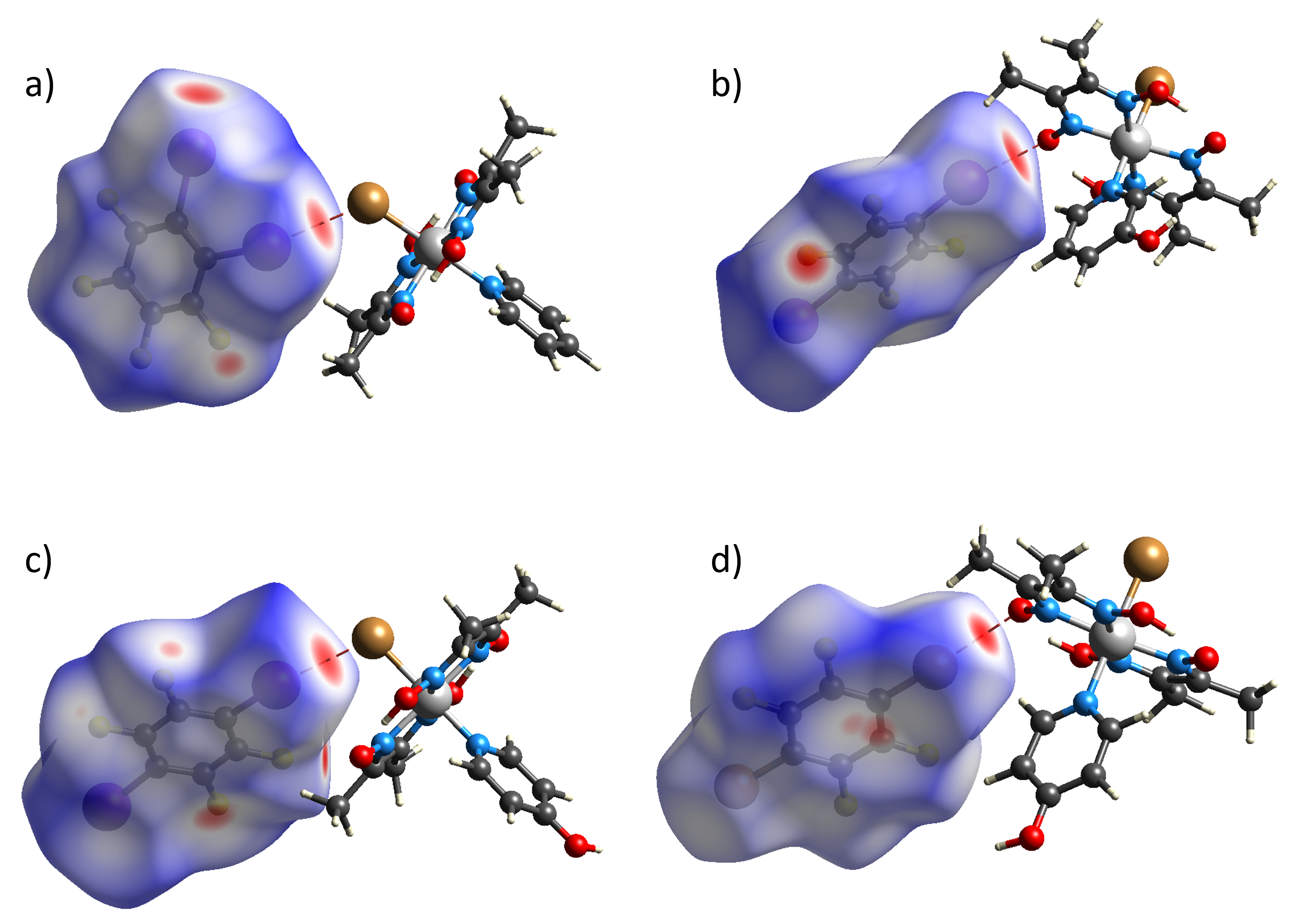


**Figure S26.** Values *d*_norm_mapped on the Hirshfeld surface of the donor molecule in (**a**) (**I**)(**12tfib**), (**b**) (**II**)_2_(**14tfbb**), (**c**) (**III**)_2_(**14tfib**) and (**d**) (**III**)_2_(**14tfbb**).


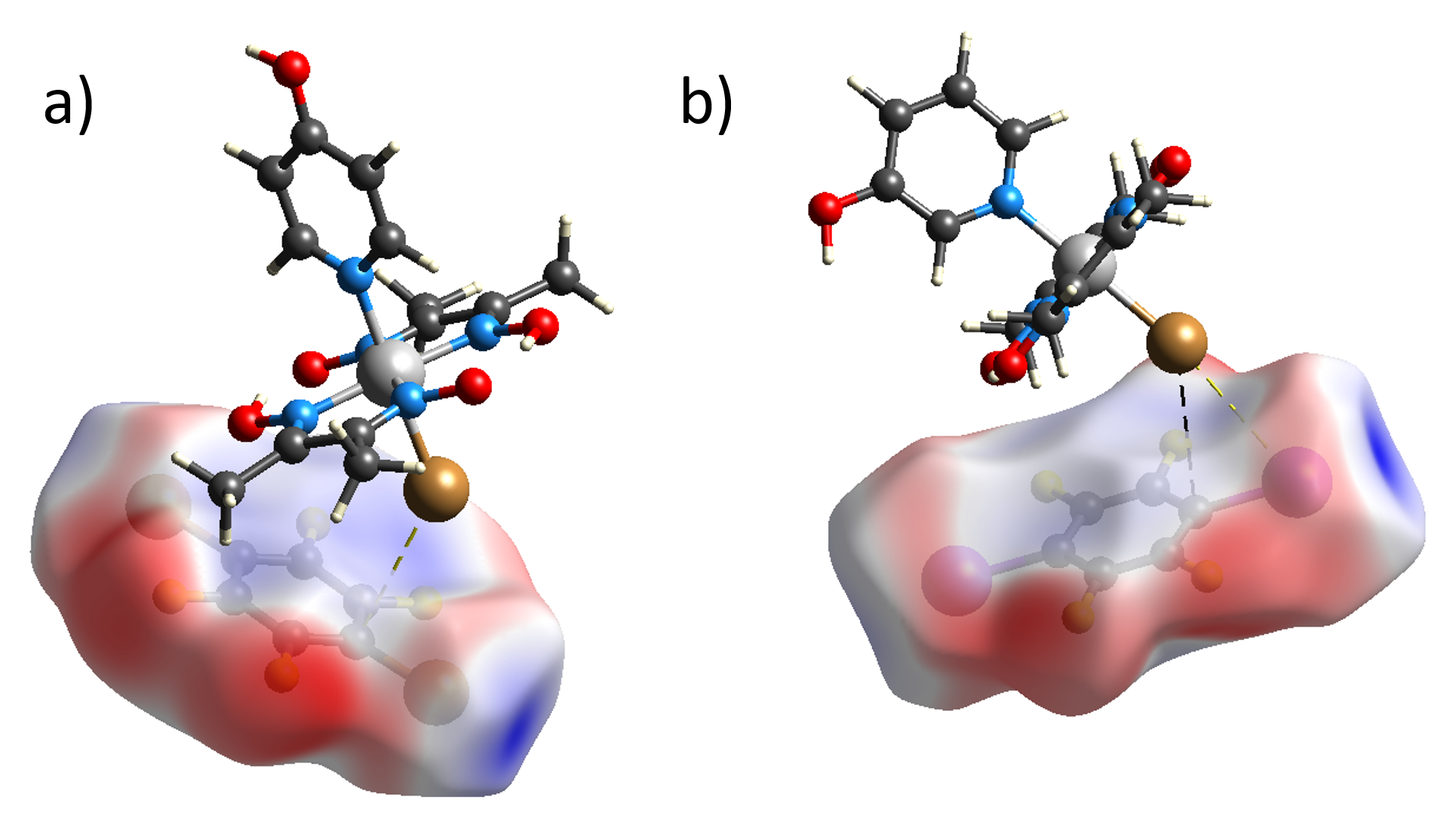


**Figure S27.** Contact between coordinated bromine atom and perfluorinated benzene ring in (**a**) (**III**)_2_(**14tfbb**) and (**b**) (**II**)_2_(**14tfbb**).


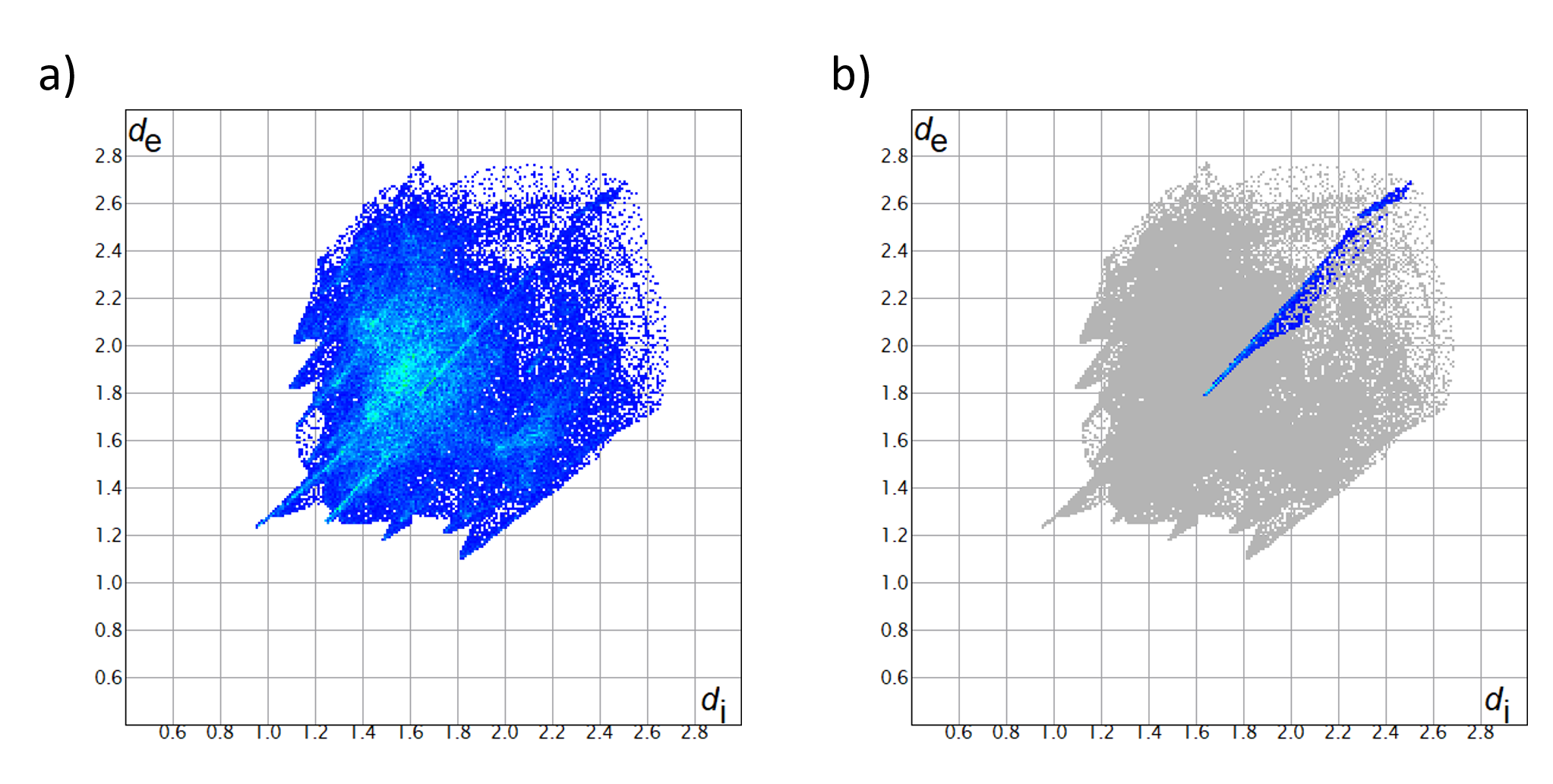


**Figure S28. (a**) Fingerprint of the Hirshfeld surface of donor molecule in cocrystal (**I**)(**12tfib**); (**b**) Pairs of the *d*_e_ and *d*_i_ values corresponding to the I‧‧‧Br halogen bond.


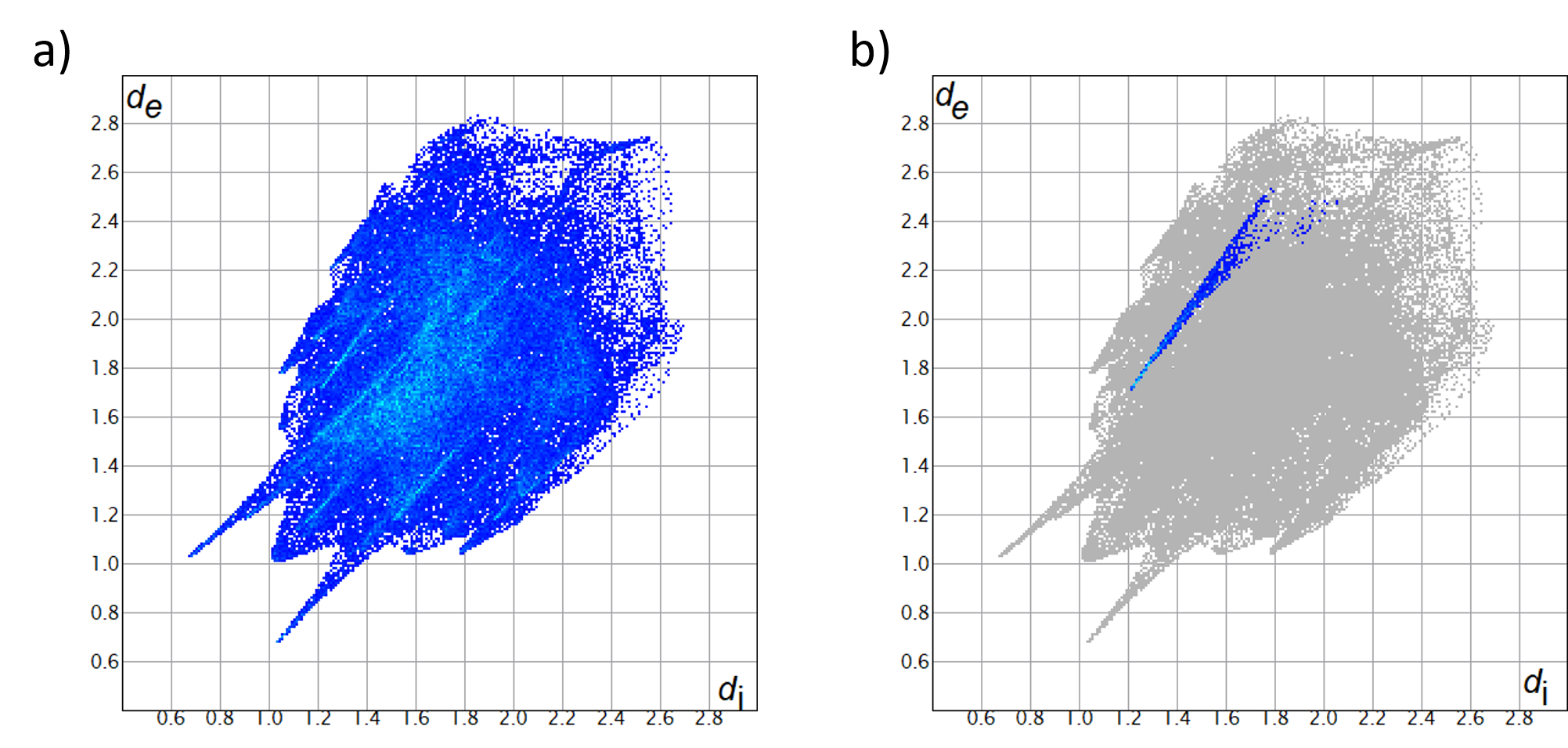


**Figure S29. (a**) Fingerprint of the Hirshfeld surface of donor molecule in cocrystal (**II**)_2_(**14tfib**); (**b**) Pairs of the *d*_e_ and *d*_i_ values corresponding to the I‧‧‧O halogen bond.


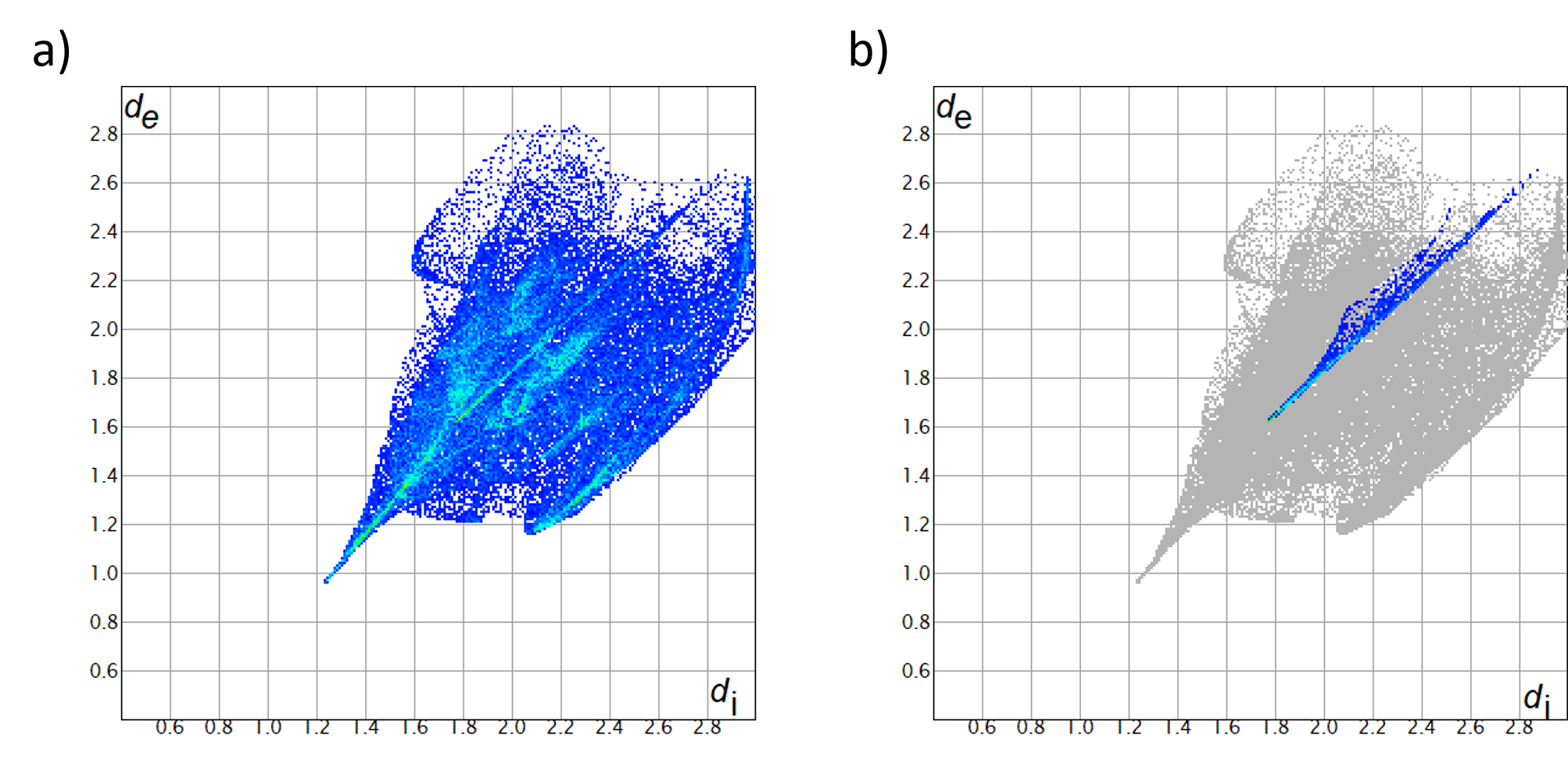


**Figure S30.** (**a**) Fingerprint of the Hirshfeld surface of donor molecule in cocrystal (**III**)_2_(**14tfib**); (**b**) Pairs of the *d*_e_ and *d*_i_ values corresponding to the I‧‧‧Br halogen bond.


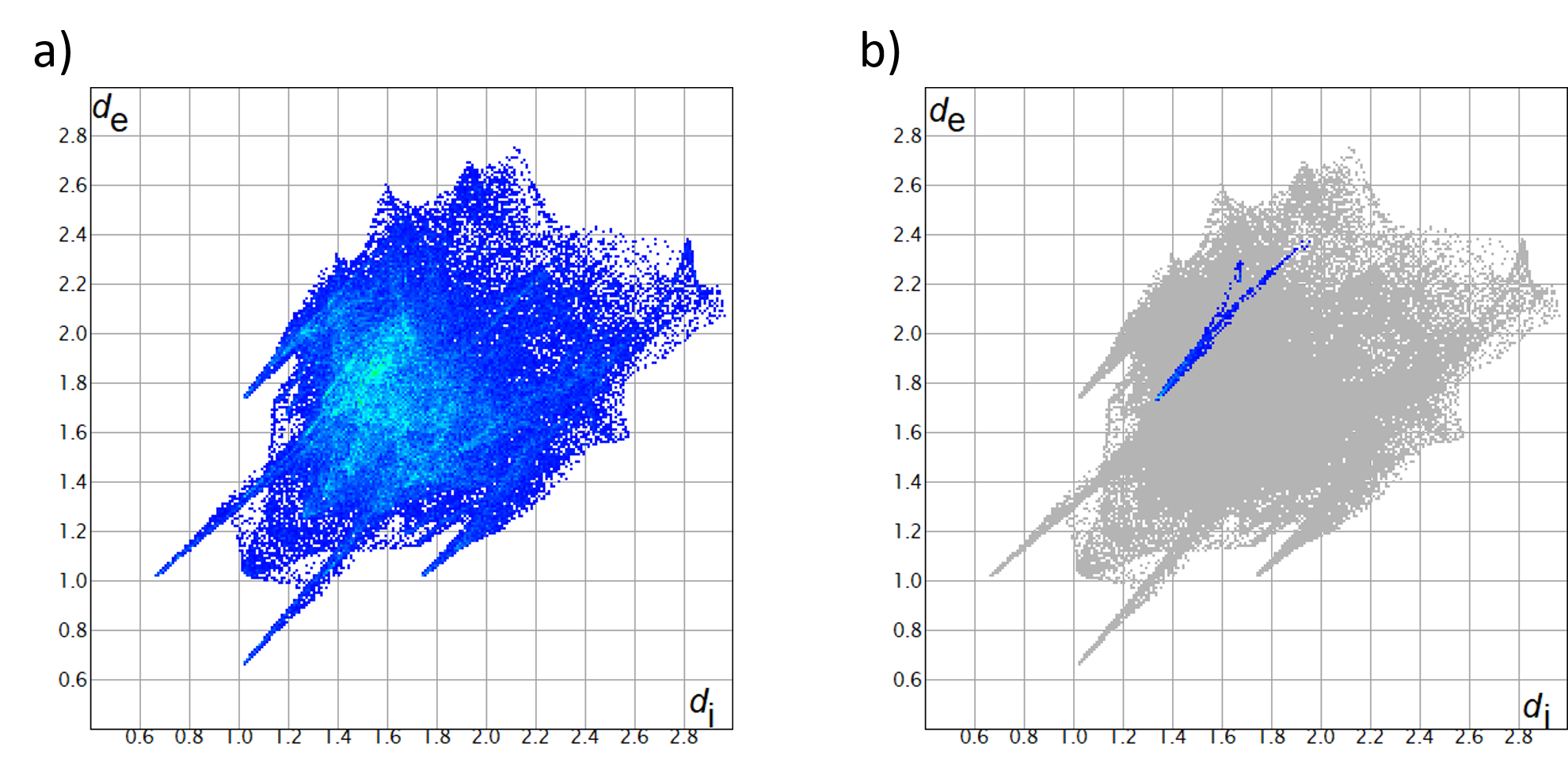


**Figure S31.** (**a**) Fingerprint of the Hirshfeld surface of donor molecule in cocrystal (**III**)_2_(**14tfbb**); (**b**) Pairs of the *d*_e_ and *d*_i_ values corresponding to the I‧‧‧O halogen bond.
