# Supplementary material for: Cobaloximes as Building Blocks in Halogen-Bonded Cocrystals
Source: Materials (Basel). 2020 May 21;13(10):2370. doi: 10.3390/ma13102370 (PMC7287722; doi:10.3390/ma13102370)

checkCIF/PLATON report

Structure factors have been supplied for datablock(s) I

THIS REPORT IS FOR GUIDANCE ONLY. IF USED AS PART OF A REVIEW PROCEDURE FOR PUBLICATION, IT SHOULD NOT REPLACE THE EXPERTISE OF AN EXPERIENCED CRYSTALLOGRAPHIC REFEREE.

No syntax errors found.   CIF dictionary   Interpreting this report

Datablock: I

```
Bond precision:  C-C = 0.0077 A          Wavelength=0.71073

Cell:            a=8.7426(6)    b=14.3293(12)     c=14.1011(9)
                 alpha=90       beta=96.923(6)    gamma=90
Temperature:     295 K

                Calculated                 Reported
Volume          1753.6(2)                  1753.6(2)
Space group     P 21/n                     P 21/n
Hall group      -P 2yn                     -P 2yn
Moiety formula  C13 H19 Br Co N5 O4        C13 H19 Br Co N5 O4
Sum formula     C13 H19 Br Co N5 O4        C13 H19 Br Co N5 O4
Mr              448.16                     448.17
Dx,g cm-3       1.697                      1.698
Z               4                          4
Mu (mm-1)       3.285                      3.284
F000            904.0                      904.0
F000'           904.57
h,k,lmax        11,18,18                   11,18,17
Nref            3824                       3776
Tmin,Tmax       0.249,0.440                0.439,1.000
Tmin'           0.209

Correction method= # Reported T Limits: Tmin=0.439 Tmax=1.000
AbsCorr = MULTI-SCAN

Data completeness= 0.987          Theta(max)= 26.998

R(reflections)= 0.0494( 2007)     wR2(reflections)= 0.1077( 3776)

S = 0.888                  Npar= 221
```

The following ALERTS were generated. Each ALERT has the format
test-name_ALERT_alert-type_alert-level.
Click on the hyperlinks for more details of the test.

Alert level B

```
PLAT910_ALERT_3_B Missing # of FCF Reflection(s) Below Theta(Min).         11 Note
```

Author Response: These reflections either have masked by the beamstop or have too high intensity (overflow), hence they have not been measured.

Alert level C

```
PLAT230_ALERT_2_C Hirshfeld Test Diff for    O3       --N3       .        7.0 s.u.
PLAT341_ALERT_3_C Low Bond Precision on  C-C Bonds ...............     0.0077 Ang.
PLAT906_ALERT_3_C Large K Value in the Analysis of Variance ......     20.257 Check
PLAT906_ALERT_3_C Large K Value in the Analysis of Variance ......      3.951 Check
PLAT906_ALERT_3_C Large K Value in the Analysis of Variance ......      2.209 Check
PLAT911_ALERT_3_C Missing FCF Refl Between Thmin & STh/L=    0.600         21 Report
```

Alert level G

```
PLAT007_ALERT_5_G Number of Unrefined Donor-H Atoms ..............          2 Report
PLAT883_ALERT_1_G No Info/Value for _atom_sites_solution_primary .     Please Do !
PLAT912_ALERT_4_G Missing # of FCF Reflections Above STh/L=  0.600         16 Note
PLAT933_ALERT_2_G Number of OMIT Records in Embedded .res File ...          3 Note
PLAT941_ALERT_3_G Average HKL Measurement Multiplicity ...........        2.2 Low
PLAT978_ALERT_2_G Number C-C Bonds with Positive Residual Density.          0 Info
```

---

0 **ALERT level A** = Most likely a serious problem - resolve or explain
1 **ALERT level B** = A potentially serious problem, consider carefully
6 **ALERT level C** = Check. Ensure it is not caused by an omission or oversight
6 **ALERT level G** = General information/check it is not something unexpected

1 ALERT type 1 CIF construction/syntax error, inconsistent or missing data
3 ALERT type 2 Indicator that the structure model may be wrong or deficient
7 ALERT type 3 Indicator that the structure quality may be low
1 ALERT type 4 Improvement, methodology, query or suggestion
1 ALERT type 5 Informative message, check

It is advisable to attempt to resolve as many as possible of the alerts in all categories. Often the minor alerts point to easily fixed oversights, errors and omissions in your CIF or refinement strategy, so attention to these fine details can be worthwhile. In order to resolve some of the more serious problems it may be necessary to carry out additional measurements or structure refinements. However, the purpose of your study may justify the reported deviations and the more serious of these should normally be commented upon in the discussion or experimental section of a paper or in the "special_details" fields of the CIF. checkCIF was carefully designed to identify outliers and unusual parameters, but every test has its limitations and alerts that are not important in a particular case may appear. Conversely, the absence of alerts does not guarantee there are no aspects of the results needing attention. It is up to the individual to critically assess their own results and, if necessary, seek expert advice.

Publication of your CIF in IUCr journals

A basic structural check has been run on your CIF. These basic checks will be run on all CIFs submitted for publication in IUCr journals (*Acta Crystallographica*, *Journal of Applied Crystallography*, *Journal of Synchrotron Radiation*); however, if you intend to submit to *Acta Crystallographica Section C* or *E* or *IUCrData*, you should make sure that full publication checks are run on the final version of your CIF prior to submission.

Publication of your CIF in other journals

Please refer to the *Notes for Authors* of the relevant journal for any special instructions relating to CIF submission.

PLATON version of 22/04/2020; check.def file version of 09/03/2020

Datablock I - ellipsoid plot

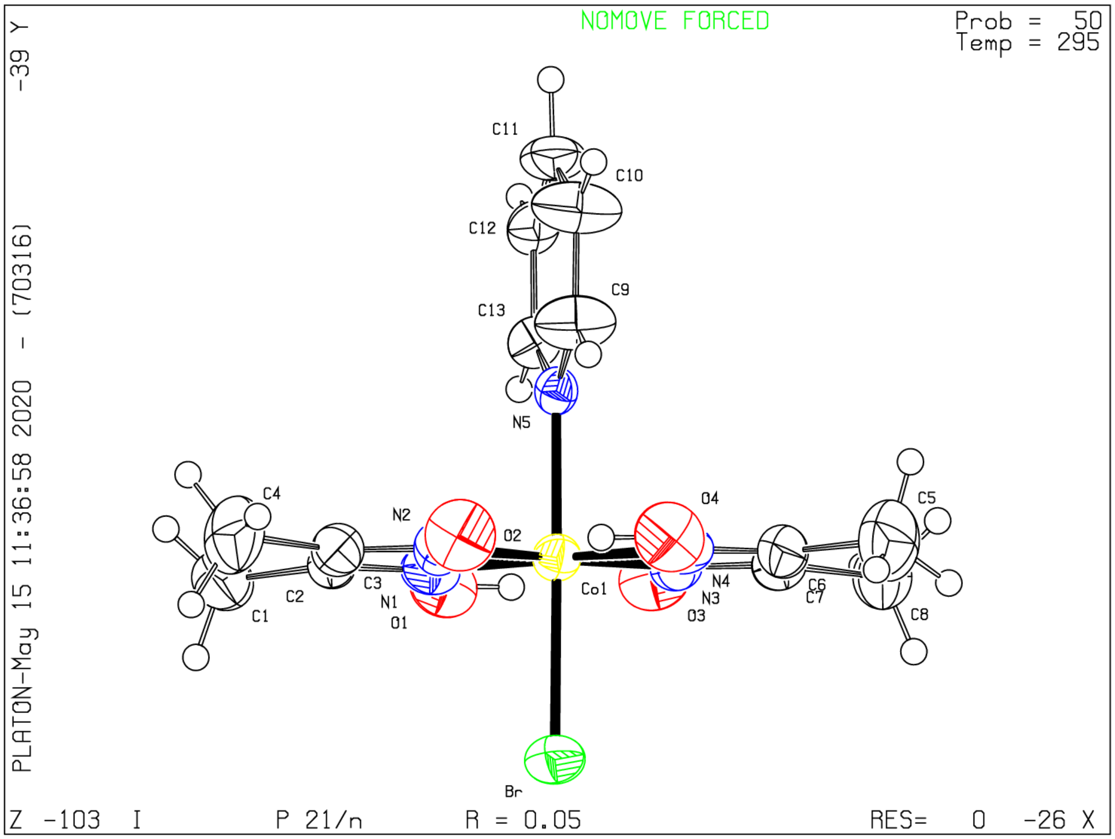

checkCIF/PLATON report

Structure factors have been supplied for datablock(s) I12tfib

THIS REPORT IS FOR GUIDANCE ONLY. IF USED AS PART OF A REVIEW PROCEDURE FOR PUBLICATION, IT SHOULD NOT REPLACE THE EXPERTISE OF AN EXPERIENCED CRYSTALLOGRAPHIC REFEREE.

No syntax errors found. CIF dictionary Interpreting this report

Datablock: I12tfib

Bond precision: C-C = 0.0169 A Wavelength=0.71073

Cell:	a=8.9048(5)	b=15.6550(5)	c=10.0166(5)
	alpha=90	beta=110.215(6)	gamma=90
Temperature:	295 K		

	Calculated	Reported
Volume	1310.35(12)	1310.35(12)
Space group	P 21	P 21
Hall group	P 2yb	P 2yb
Moiety formula	C13 H19 Br Co N5 O4, C6 F4 I2	C13 H19 Br Co N5 O4, C6 F4 I2
Sum formula	C19 H19 Br Co F4 I2 N5 O4	C19 H19 Br Co F4 I2 N5 O4
Mr	850.02	850.03
Dx,g cm-3	2.154	2.154
Z	2	2
Mu (mm-1)	4.599	4.599
F000	808.0	808.0
F000’	806.63	
h,k,lmax	10,18,11	10,18,11
Nref	4626[2405]	4575
Tmin,Tmax	0.208,0.317	0.454,1.000
Tmin’	0.160	

Correction method= # Reported T Limits: Tmin=0.454 Tmax=1.000
AbsCorr = MULTI-SCAN

Data completeness= 1.90/0.99 Theta(max)= 24.996

R(reflections)= 0.0345(3622) wR2(reflections)= 0.0732(4575)

S = 0.896 Npar= 329

The following ALERTS were generated. Each ALERT has the format

test-name_ALERT_alert-type_alert-level.

Click on the hyperlinks for more details of the test.

Alert level B

```
PLAT910_ALERT_3_B Missing # of FCF Reflection(s) Below Theta(Min).        11 Note
```

Author Response: These reflections either have masked by the beamstop or have too high intensity (overflow), hence they have not been measured.

Alert level C

```
PLAT090_ALERT_3_C Poor Data / Parameter Ratio (Zmax > 18) ........       7.27 Note
PLAT334_ALERT_2_C Small Aver. Benzene C-C Dist C020     -C029             1.37 Ang.
PLAT342_ALERT_3_C Low Bond Precision on  C-C Bonds ...............     0.01694 Ang.
PLAT911_ALERT_3_C Missing FCF Refl Between Thmin & STh/L=    0.595           3 Report
```

Alert level G

```
PLAT007_ALERT_5_G Number of Unrefined Donor-H Atoms ..............           2 Report
PLAT153_ALERT_1_G The s.u.'s on the Cell Axes   are Equal ..(Note)      0.0005 Ang.
PLAT431_ALERT_2_G Short Inter HL..A Contact  I002     ..O3       .        3.13 Ang.
                                              1-x,1/2+y,1-z  =      2_656 Check
PLAT434_ALERT_2_G Short Inter HL..HL Contact I001     ..Br03              3.44 Ang.
                                                 1+x,y,-1+z  =      1_654 Check
PLAT720_ALERT_4_G Number of Unusual/Non-Standard Labels ..........          40 Note
PLAT883_ALERT_1_G No Info/Value for _atom_sites_solution_primary .      Please Do !
PLAT909_ALERT_3_G Percentage of I>2sig(I) Data at Theta(Max) Still         48% Note
PLAT941_ALERT_3_G Average HKL Measurement Multiplicity ...........         3.8 Low
PLAT978_ALERT_2_G Number C-C Bonds with Positive Residual Density.           0 Info
```

---

0 **ALERT level A** = Most likely a serious problem - resolve or explain
1 **ALERT level B** = A potentially serious problem, consider carefully
4 **ALERT level C** = Check. Ensure it is not caused by an omission or oversight
9 **ALERT level G** = General information/check it is not something unexpected

2 ALERT type 1 CIF construction/syntax error, inconsistent or missing data
4 ALERT type 2 Indicator that the structure model may be wrong or deficient
6 ALERT type 3 Indicator that the structure quality may be low
1 ALERT type 4 Improvement, methodology, query or suggestion
1 ALERT type 5 Informative message, check

It is advisable to attempt to resolve as many as possible of the alerts in all categories. Often the minor alerts point to easily fixed oversights, errors and omissions in your CIF or refinement strategy, so attention to these fine details can be worthwhile. In order to resolve some of the more serious problems it may be necessary to carry out additional measurements or structure refinements. However, the purpose of your study may justify the reported deviations and the more serious of these should normally be commented upon in the discussion or experimental section of a paper or in the "special_details" fields of the CIF. checkCIF was carefully designed to identify outliers and unusual parameters, but every test has its limitations and alerts that are not important in a particular case may appear. Conversely, the absence of alerts does not guarantee there are no aspects of the results needing attention. It is up to the individual to critically assess their own results and, if necessary, seek expert advice.

Publication of your CIF in IUCr journals

A basic structural check has been run on your CIF. These basic checks will be run on all CIFs submitted for publication in IUCr journals (*Acta Crystallographica*, *Journal of Applied Crystallography*, *Journal of Synchrotron Radiation*); however, if you intend to submit to *Acta Crystallographica Section C* or *E* or *IUCrData*, you should make sure that full publication checks are run on the final version of your CIF prior to submission.

Publication of your CIF in other journals

Please refer to the *Notes for Authors* of the relevant journal for any special instructions relating to CIF submission.

PLATON version of 22/04/2020; check.def file version of 09/03/2020

Datablock I12tfib - ellipsoid plot

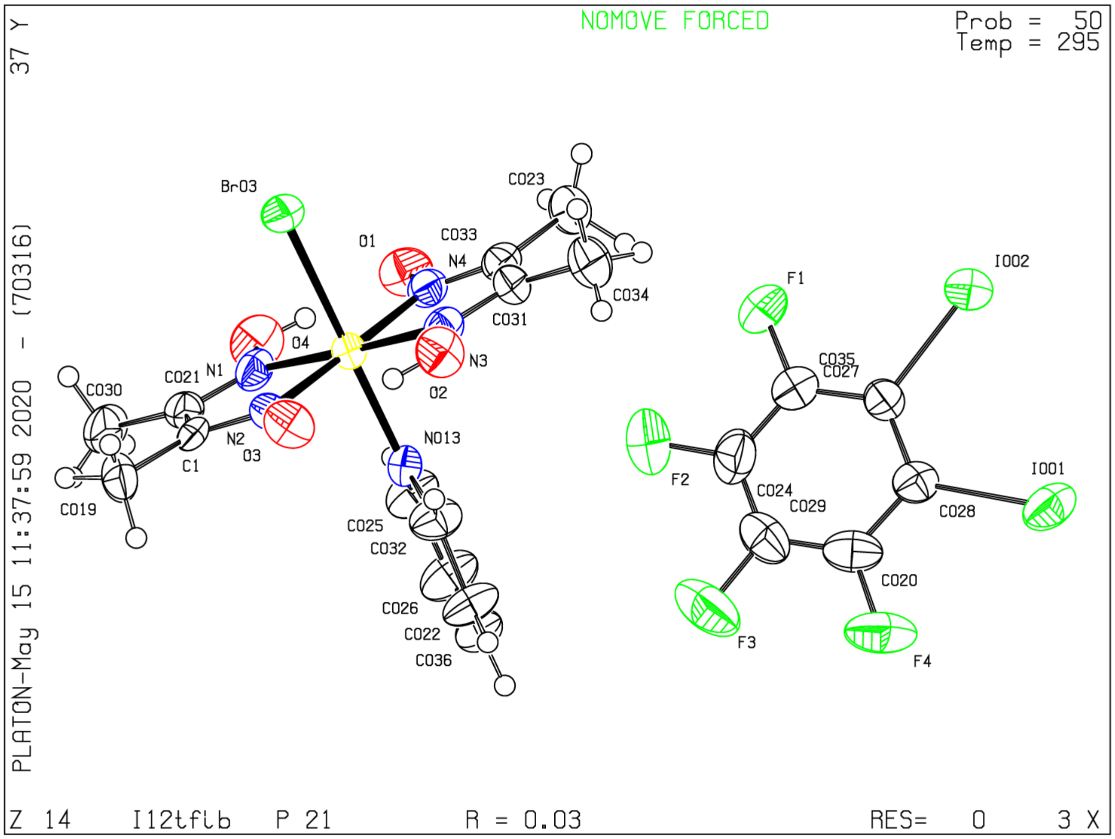

checkCIF/PLATON report

Structure factors have been supplied for datablock(s) II

THIS REPORT IS FOR GUIDANCE ONLY. IF USED AS PART OF A REVIEW PROCEDURE FOR PUBLICATION, IT SHOULD NOT REPLACE THE EXPERTISE OF AN EXPERIENCED CRYSTALLOGRAPHIC REFEREE.

No syntax errors found. CIF dictionary Interpreting this report

Datablock: II

```
Bond precision:  C-C = 0.0070 A          Wavelength=0.71073

Cell:            a=8.7876(3)     b=14.8321(6)       c=13.9188(4)
                 alpha=90        beta=94.610(3)     gamma=90
Temperature:     295 K

                Calculated                Reported
Volume          1808.29(11)               1808.29(11)
Space group     P n                       P n
Hall group      P -2yac                   P -2yac
Moiety formula  C13 H19 Br Co N5 O5       C13 H19 Br Co N5 O5
Sum formula     C13 H19 Br Co N5 O5       C13 H19 Br Co N5 O5
Mr              464.16                    464.17
Dx,g cm-3       1.705                     1.705
Z               4                         4
Mu (mm-1)       3.192                     3.192
F000            936.0                     936.0
F000'           936.61
h,k,lmax        10,17,16                  10,17,16
Nref            6383[ 3200]               6353
Tmin,Tmax       0.281,0.384               0.370,1.000
Tmin'           0.260

Correction method= # Reported T Limits: Tmin=0.370 Tmax=1.000
AbsCorr = MULTI-SCAN

Data completeness= 1.99/1.00      Theta(max)= 24.999

R(reflections)= 0.0211( 5750)     wR2(reflections)= 0.0473( 6353)

S = 0.930                 Npar= 452
```

```
The following ALERTS were generated. Each ALERT has the format
       test-name_ALERT_alert-type_alert-level.
Click on the hyperlinks for more details of the test.
```

Alert level B

```
PLAT109_ALERT_2_B Twinning Matrix is inverted Laue group operation          ? Check
PLAT910_ALERT_3_B Missing # of FCF Reflection(s) Below Theta(Min).         12 Note
```

Author Response: These reflections either have masked by the beamstop or have too high intensity (overflow), hence they have not been measured.

Alert level C

```
PLAT090_ALERT_3_C Poor Data / Parameter Ratio (Zmax > 18) ........       7.05 Note
PLAT341_ALERT_3_C Low Bond Precision on  C-C Bonds ..............    0.00705 Ang.
PLAT413_ALERT_2_C Short Inter XH3 .. XHn     H8B      ..H14B     .       2.09 Ang.
                                          -1/2+x,1-y,-1/2+z  =      2_464 Check
```

Alert level G

```
PLAT007_ALERT_5_G Number of Unrefined Donor-H Atoms ..............          6 Report
PLAT380_ALERT_4_G Incorrectly? Oriented X(sp2)-Methyl Moiety .....         C1 Check
PLAT380_ALERT_4_G Incorrectly? Oriented X(sp2)-Methyl Moiety .....         C4 Check
PLAT380_ALERT_4_G Incorrectly? Oriented X(sp2)-Methyl Moiety .....         C5 Check
PLAT380_ALERT_4_G Incorrectly? Oriented X(sp2)-Methyl Moiety .....         C8 Check
PLAT380_ALERT_4_G Incorrectly? Oriented X(sp2)-Methyl Moiety .....        C14 Check
PLAT380_ALERT_4_G Incorrectly? Oriented X(sp2)-Methyl Moiety .....        C17 Check
PLAT380_ALERT_4_G Incorrectly? Oriented X(sp2)-Methyl Moiety .....        C18 Check
PLAT380_ALERT_4_G Incorrectly? Oriented X(sp2)-Methyl Moiety .....        C21 Check
PLAT870_ALERT_4_G ALERTS Related to Twinning Effects Suppressed ..          ! Info
PLAT883_ALERT_1_G No Info/Value for _atom_sites_solution_primary .     Please Do !
PLAT909_ALERT_3_G Percentage of I>2sig(I) Data at Theta(Max) Still        74% Note
PLAT913_ALERT_3_G Missing # of Very Strong Reflections in FCF ....          1 Note
PLAT916_ALERT_2_G Hooft y and Flack x Parameter Values Differ by .       0.58 Check
PLAT931_ALERT_5_G CIFcalcFCF  Twin Law ( 0 1 0)        Est.d BASF        0.57 Check
PLAT933_ALERT_2_G Number of OMIT Records in Embedded .res File ...          2 Note
```

```
   0 ALERT level A = Most likely a serious problem - resolve or explain
   2 ALERT level B = A potentially serious problem, consider carefully
   3 ALERT level C = Check. Ensure it is not caused by an omission or oversight
  16 ALERT level G = General information/check it is not something unexpected

   1 ALERT type 1 CIF construction/syntax error, inconsistent or missing data
   4 ALERT type 2 Indicator that the structure model may be wrong or deficient
   5 ALERT type 3 Indicator that the structure quality may be low
   9 ALERT type 4 Improvement, methodology, query or suggestion
   2 ALERT type 5 Informative message, check
```

It is advisable to attempt to resolve as many as possible of the alerts in all categories. Often the minor alerts point to easily fixed oversights, errors and omissions in your CIF or refinement strategy, so attention to these fine details can be worthwhile. In order to resolve some of the more serious problems it may be necessary to carry out additional measurements or structure refinements. However, the purpose of your study may justify the reported deviations and the more serious of these should normally be commented upon in the discussion or experimental section of a paper or in the "special_details" fields of the CIF. checkCIF was carefully designed to identify outliers and unusual parameters, but every test has its limitations and alerts that are not important in a particular case may appear. Conversely, the absence of alerts does not guarantee there are no aspects of the results needing attention. It is up to the individual to critically assess their own results and, if necessary, seek expert advice.

Publication of your CIF in IUCr journals

A basic structural check has been run on your CIF. These basic checks will be run on all CIFs submitted for publication in IUCr journals (*Acta Crystallographica*, *Journal of Applied Crystallography*, *Journal of Synchrotron Radiation*); however, if you intend to submit to *Acta Crystallographica Section C* or *E* or *IUCrData*, you should make sure that full publication checks are run on the final version of your CIF prior to submission.

Publication of your CIF in other journals

Please refer to the *Notes for Authors* of the relevant journal for any special instructions relating to CIF submission.

PLATON version of 22/04/2020; check.def file version of 09/03/2020

Datablock II - ellipsoid plot

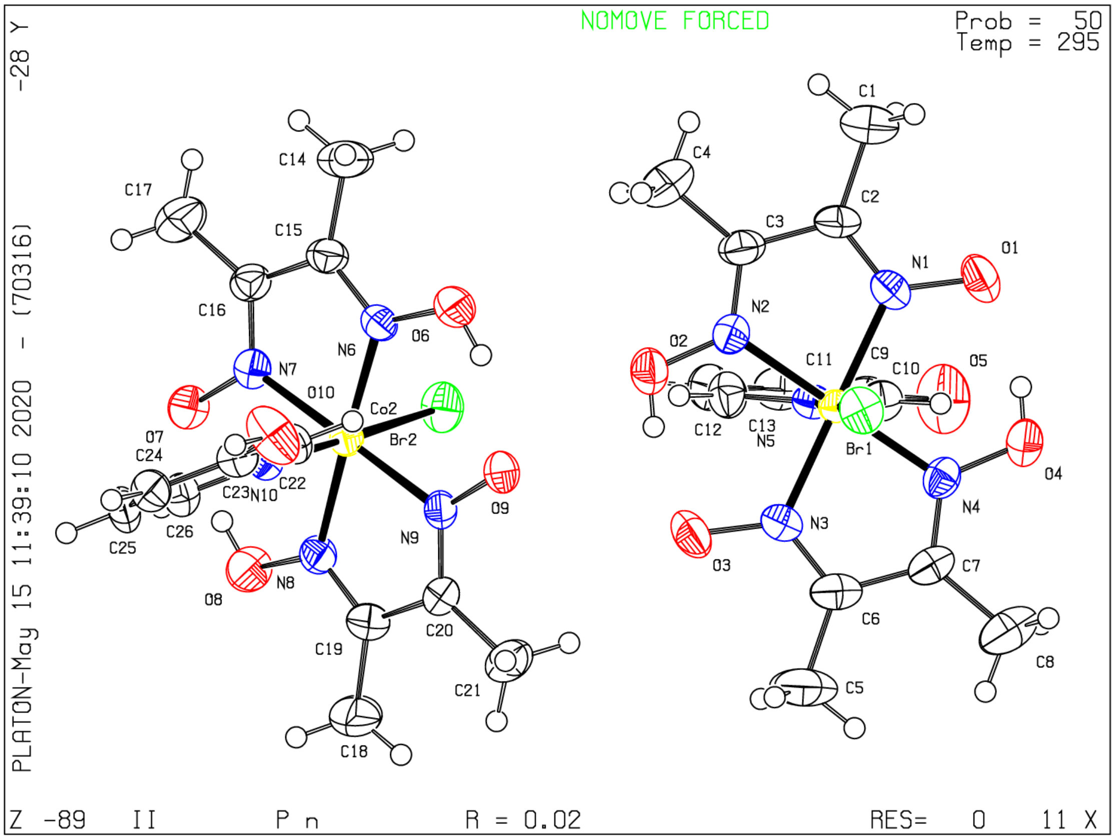

checkCIF/PLATON report

Structure factors have been supplied for datablock(s) II14tfib

THIS REPORT IS FOR GUIDANCE ONLY. IF USED AS PART OF A REVIEW PROCEDURE FOR PUBLICATION, IT SHOULD NOT REPLACE THE EXPERTISE OF AN EXPERIENCED CRYSTALLOGRAPHIC REFEREE.

No syntax errors found. CIF dictionary Interpreting this report

Datablock: II14tfib

Bond precision: C-C = 0.0105 A Wavelength=0.71073

Cell:	a=8.3528(8)	b=22.5657(18)	c=11.9617(8)
	alpha=90	beta=91.460(7)	gamma=90
Temperature:	295 K		

	Calculated	Reported
Volume	2253.9(3)	2253.9(3)
Space group	P 21/n	P 21/n
Hall group	-P 2yn	-P 2yn
Moiety formula	C13 H19 Br Co N5 O5, 0.5(C6 F4 I2)	C13 H19 Br Co N5 O5, 0.5(C6 F4 I2)
Sum formula	C16 H19 Br Co F2 I N5 O5	C16 H19 Br Co F2 I N5 O5
Mr	665.09	665.10
Dx,g cm-3	1.960	1.960
Z	4	4
Mu (mm-1)	3.957	3.957
F000	1292.0	1292.0
F000’	1290.95	
h,k,lmax	9,26,14	9,26,14
Nref	3965	3937
Tmin,Tmax	0.233,0.317	0.653,1.000
Tmin’	0.196	

Correction method= # Reported T Limits: Tmin=0.653 Tmax=1.000
AbsCorr = MULTI-SCAN

Data completeness= 0.993 Theta(max)= 25.000

R(reflections)= 0.0506(2561) wR2(reflections)= 0.1231(3937)

S = 0.969 Npar= 280

The following ALERTS were generated. Each ALERT has the format
test-name_ALERT_alert-type_alert-level.
Click on the hyperlinks for more details of the test.

Alert level B

```
PLAT910_ALERT_3_B Missing # of FCF Reflection(s) Below Theta(Min).        26 Note
```

Author Response: These reflections either have masked by the beamstop or have too high intensity (overflow), hence they have not been measured.

Alert level C

```
PLAT334_ALERT_2_C Small Aver. Benzene C-C Dist C14      -C16_a          1.37 Ang.
PLAT342_ALERT_3_C Low Bond Precision on  C-C Bonds ...............    0.01046 Ang.
PLAT906_ALERT_3_C Large K Value in the Analysis of Variance ......      2.211 Check
PLAT911_ALERT_3_C Missing FCF Refl Between Thmin & STh/L=    0.595          2 Report
```

Alert level G

```
PLAT007_ALERT_5_G Number of Unrefined Donor-H Atoms ..............          3 Report
PLAT380_ALERT_4_G Incorrectly? Oriented X(sp2)-Methyl Moiety .....         C1 Check
PLAT380_ALERT_4_G Incorrectly? Oriented X(sp2)-Methyl Moiety .....         C4 Check
PLAT380_ALERT_4_G Incorrectly? Oriented X(sp2)-Methyl Moiety .....         C5 Check
PLAT380_ALERT_4_G Incorrectly? Oriented X(sp2)-Methyl Moiety .....         C8 Check
PLAT431_ALERT_2_G Short Inter HL..A Contact  I1       ..O4       .       2.94 Ang.
                                         1/2+x,1/2-y,-1/2+z  =      4_665 Check
PLAT883_ALERT_1_G No Info/Value for _atom_sites_solution_primary .     Please Do !
PLAT909_ALERT_3_G Percentage of I>2sig(I) Data at Theta(Max) Still        37% Note
PLAT933_ALERT_2_G Number of OMIT Records in Embedded .res File ...          7 Note
PLAT941_ALERT_3_G Average HKL Measurement Multiplicity ...........        3.3 Low
PLAT978_ALERT_2_G Number C-C Bonds with Positive Residual Density.          0 Info
```

---

0 **ALERT level A** = Most likely a serious problem - resolve or explain
1 **ALERT level B** = A potentially serious problem, consider carefully
4 **ALERT level C** = Check. Ensure it is not caused by an omission or oversight
11 **ALERT level G** = General information/check it is not something unexpected

1 ALERT type 1 CIF construction/syntax error, inconsistent or missing data
4 ALERT type 2 Indicator that the structure model may be wrong or deficient
6 ALERT type 3 Indicator that the structure quality may be low
4 ALERT type 4 Improvement, methodology, query or suggestion
1 ALERT type 5 Informative message, check

It is advisable to attempt to resolve as many as possible of the alerts in all categories. Often the minor alerts point to easily fixed oversights, errors and omissions in your CIF or refinement strategy, so attention to these fine details can be worthwhile. In order to resolve some of the more serious problems it may be necessary to carry out additional measurements or structure refinements. However, the purpose of your study may justify the reported deviations and the more serious of these should normally be commented upon in the discussion or experimental section of a paper or in the "special_details" fields of the CIF. checkCIF was carefully designed to identify outliers and unusual parameters, but every test has its limitations and alerts that are not important in a particular case may appear. Conversely, the absence of alerts does not guarantee there are no aspects of the results needing attention. It is up to the individual to critically assess their own results and, if necessary, seek expert advice.

Publication of your CIF in IUCr journals

A basic structural check has been run on your CIF. These basic checks will be run on all CIFs submitted for publication in IUCr journals (*Acta Crystallographica*, *Journal of Applied Crystallography*, *Journal of Synchrotron Radiation*); however, if you intend to submit to *Acta Crystallographica Section C* or *E* or *IUCrData*, you should make sure that full publication checks are run on the final version of your CIF prior to submission.

Publication of your CIF in other journals

Please refer to the *Notes for Authors* of the relevant journal for any special instructions relating to CIF submission.

PLATON version of 22/04/2020; check.def file version of 09/03/2020

Datablock II14tfib - ellipsoid plot

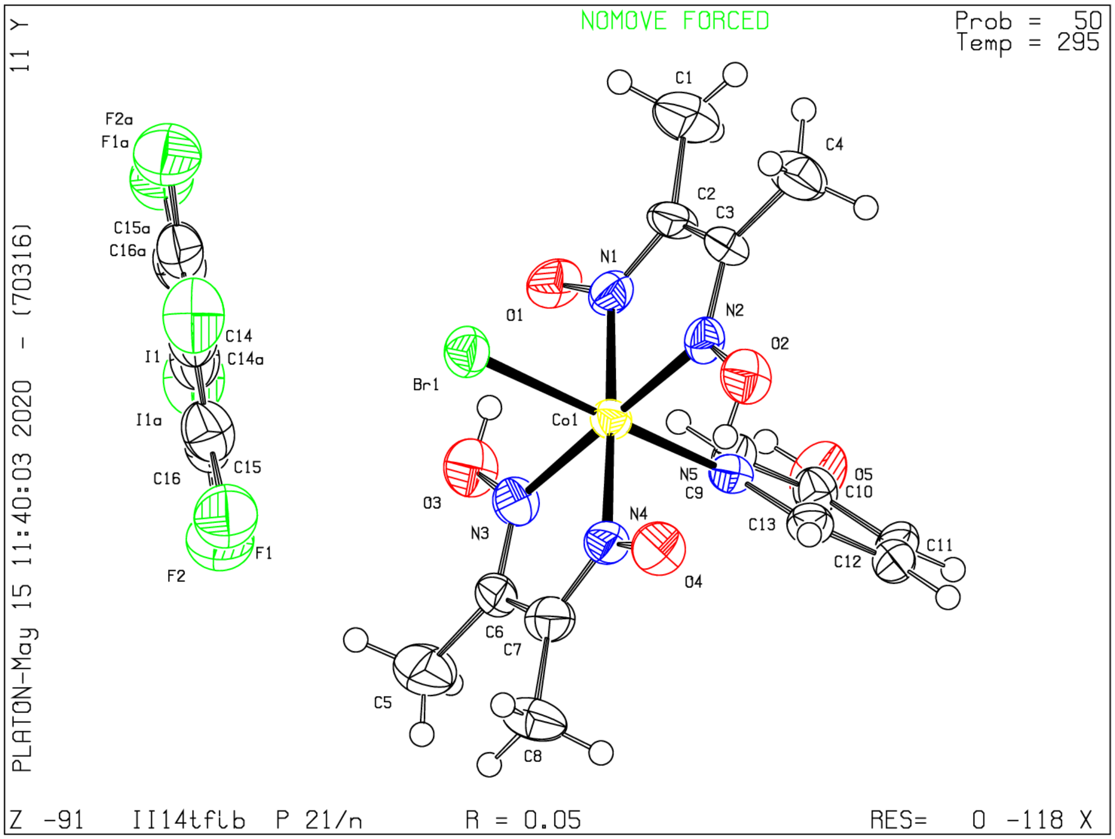

checkCIF/PLATON report

Structure factors have been supplied for datablock(s) III14tfbb

THIS REPORT IS FOR GUIDANCE ONLY. IF USED AS PART OF A REVIEW PROCEDURE FOR PUBLICATION, IT SHOULD NOT REPLACE THE EXPERTISE OF AN EXPERIENCED CRYSTALLOGRAPHIC REFEREE.

No syntax errors found. CIF dictionary Interpreting this report

Datablock: III14tfbb

```
Bond precision:  C-C = 0.0078 A          Wavelength=0.71073

Cell:            a=8.9682(4)     b=18.2906(10)     c=26.5773(9)
                 alpha=90        beta=90           gamma=90
Temperature:     295 K
```

	Calculated	Reported
Volume	4359.6(3)	4359.6(3)
Space group	P b c a	P b c a
Hall group	-P 2ac 2ab	-P 2ac 2ab
Moiety formula	C13 H19 Br Co N5 O5, 0.5(C6 Br2 F4)	C13 H19 Br Co N5 O5, 0.5(C6 Br2 F4)
Sum formula	C16 H19 Br2 Co F2 N5 O5	C32 H38 Br4 Co2 F4 N10 O10
Mr	618.09	1236.22
Dx,g cm-3	1.883	1.883
Z	8	4
Mu (mm-1)	4.509	4.509
F000	2440.0	2440.0
F000’	2439.43	
h,k,lmax	11,23,33	11,23,33
Nref	4761	4587
Tmin,Tmax	0.115,0.157	0.397,1.000
Tmin’	0.087	

```
Correction method= # Reported T Limits: Tmin=0.397 Tmax=1.000
AbsCorr = MULTI-SCAN

Data completeness= 0.963          Theta(max)= 26.998

R(reflections)= 0.0535( 2478)     wR2(reflections)= 0.1102( 4587)

S = 0.904                   Npar= 284
```

```
The following ALERTS were generated. Each ALERT has the format
       test-name_ALERT_alert-type_alert-level.
Click on the hyperlinks for more details of the test.
```

Alert level B

```
PLAT910_ALERT_3_B Missing # of FCF Reflection(s) Below Theta(Min).         12 Note
```

Author Response: These reflections either have masked by the beamstop or have too high intensity (overflow), hence they have not been measured.

Alert level C

```
PLAT334_ALERT_2_C Small Aver. Benzene C-C Dist C14      -C16_a           1.37 Ang.
PLAT341_ALERT_3_C Low Bond Precision on  C-C Bonds ...............    0.00785 Ang.
PLAT431_ALERT_2_C Short Inter HL..A Contact  Br2      ..O4       .       3.08 Ang.
                                            1/2+x,1/2-y,2-z  =      3_557 Check
PLAT906_ALERT_3_C Large K Value in the Analysis of Variance ......     25.860 Check
PLAT906_ALERT_3_C Large K Value in the Analysis of Variance ......      5.124 Check
PLAT906_ALERT_3_C Large K Value in the Analysis of Variance ......      2.117 Check
PLAT911_ALERT_3_C Missing FCF Refl Between Thmin & STh/L=    0.600         66 Report
```

Alert level G

```
FORMU01_ALERT_1_G  There is a discrepancy between the atom counts in the
            _chemical_formula_sum and _chemical_formula_moiety. This is
            usually due to the moiety formula being in the wrong format.
            Atom count from _chemical_formula_sum:   C32 H38 Br4 Co2 F4 N10 O10
            Atom count from _chemical_formula_moiety:C16 H19 Br2 Co1 F2 N5 O5
PLAT007_ALERT_5_G Number of Unrefined Donor-H Atoms .............           3 Report
PLAT045_ALERT_1_G Calculated and Reported Z Differ by a Factor ...       2.00 Check
PLAT883_ALERT_1_G No Info/Value for _atom_sites_solution_primary .     Please Do !
PLAT912_ALERT_4_G Missing # of FCF Reflections Above STh/L=  0.600         96 Note
PLAT941_ALERT_3_G Average HKL Measurement Multiplicity ...........        3.1 Low
PLAT978_ALERT_2_G Number C-C Bonds with Positive Residual Density.          0 Info
```

```
   0 ALERT level A = Most likely a serious problem - resolve or explain
   1 ALERT level B = A potentially serious problem, consider carefully
   7 ALERT level C = Check. Ensure it is not caused by an omission or oversight
   7 ALERT level G = General information/check it is not something unexpected

   3 ALERT type 1 CIF construction/syntax error, inconsistent or missing data
   3 ALERT type 2 Indicator that the structure model may be wrong or deficient
   7 ALERT type 3 Indicator that the structure quality may be low
   1 ALERT type 4 Improvement, methodology, query or suggestion
   1 ALERT type 5 Informative message, check
```

It is advisable to attempt to resolve as many as possible of the alerts in all categories. Often the minor alerts point to easily fixed oversights, errors and omissions in your CIF or refinement strategy, so attention to these fine details can be worthwhile. In order to resolve some of the more serious problems it may be necessary to carry out additional measurements or structure refinements. However, the purpose of your study may justify the reported deviations and the more serious of these should normally be commented upon in the discussion or experimental section of a paper or in the "special_details" fields of the CIF. checkCIF was carefully designed to identify outliers and unusual parameters, but every test has its limitations and alerts that are not important in a particular case may appear. Conversely, the absence of alerts does not guarantee there are no aspects of the results needing attention. It is up to the individual to critically assess their own results and, if necessary, seek expert advice.

Publication of your CIF in IUCr journals

A basic structural check has been run on your CIF. These basic checks will be run on all CIFs submitted for publication in IUCr journals (*Acta Crystallographica*, *Journal of Applied Crystallography*, *Journal of Synchrotron Radiation*); however, if you intend to submit to *Acta Crystallographica Section C* or *E* or *IUCrData*, you should make sure that full publication checks are run on the final version of your CIF prior to submission.

Publication of your CIF in other journals

Please refer to the *Notes for Authors* of the relevant journal for any special instructions relating to CIF submission.

PLATON version of 22/04/2020; check.def file version of 09/03/2020

Datablock III14tfbb - ellipsoid plot

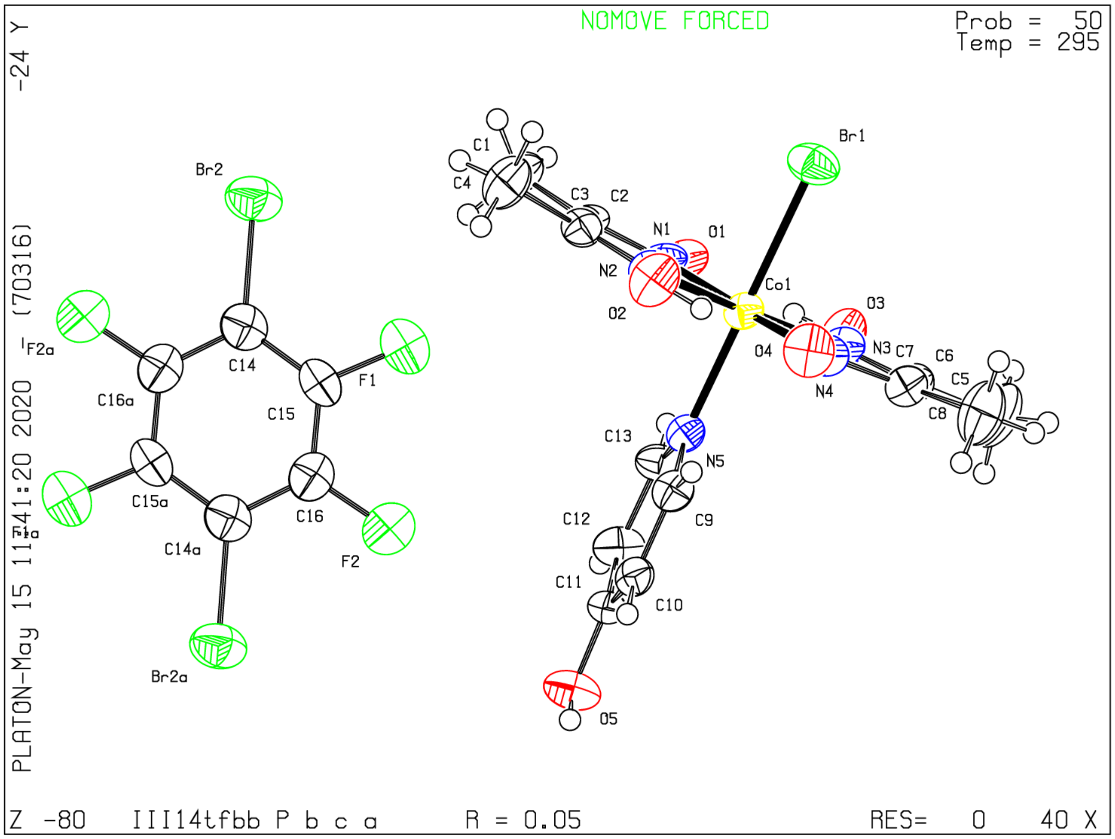

checkCIF/PLATON report

Structure factors have been supplied for datablock(s) III14tfib

THIS REPORT IS FOR GUIDANCE ONLY. IF USED AS PART OF A REVIEW PROCEDURE FOR PUBLICATION, IT SHOULD NOT REPLACE THE EXPERTISE OF AN EXPERIENCED CRYSTALLOGRAPHIC REFEREE.

No syntax errors found. CIF dictionary Interpreting this report

Datablock: III14tfib

Bond precision: C-C = 0.0093 A Wavelength=0.71073

Cell:	a=10.7618(9)	b=8.8634(6)	c=28.628(6)
	alpha=90	beta=90.506(11)	gamma=90
Temperature:	295 K		

	Calculated	Reported
Volume	2730.6(6)	2730.6(6)
Space group	P 21/n	P 21/n
Hall group	-P 2yn	-P 2yn
Moiety formula	C13 H19 Br Co N5 O5, C6 F4 I2	C13 H19 Br Co N5 O5, C6 F4 I2
Sum formula	C19 H19 Br Co F4 I2 N5 O5	C19 H19 Br Co F4 I2 N5 O5
Mr	866.02	866.03
Dx,g cm-3	2.107	2.107
Z	4	4
Mu (mm-1)	4.419	4.419
F000	1648.0	1648.0
F000’	1645.29	
h,k,lmax	12,10,34	12,10,34
Nref	4815	4517
Tmin,Tmax	0.126,0.137	0.571,1.000
Tmin’	0.080	

Correction method= # Reported T Limits: Tmin=0.571 Tmax=1.000
AbsCorr = MULTI-SCAN

Data completeness= 0.938 Theta(max)= 24.997

R(reflections)= 0.0350(2294) wR2(reflections)= 0.0627(4517)

S = 0.722 Npar= 338

The following ALERTS were generated. Each ALERT has the format
test-name_ALERT_alert-type_alert-level.
Click on the hyperlinks for more details of the test.

Alert level B

```
PLAT910_ALERT_3_B Missing # of FCF Reflection(s) Below Theta(Min).         14 Note
```

Author Response: These reflections either have masked by the beamstop or have too high intensity (overflow), hence they have not been measured.

```
PLAT911_ALERT_3_B Missing FCF Refl Between Thmin & STh/L=    0.595        284 Report
```

Alert level C

```
GOODF01_ALERT_2_C  The least squares goodness of fit parameter lies
            outside the range 0.80 <> 2.00
            Goodness of fit given =      0.722
PLAT029_ALERT_3_C _diffrn_measured_fraction_theta_full value Low .      0.968 Why?
PLAT242_ALERT_2_C Low    'MainMol' Ueq as Compared to Neighbors of        C17 Check
PLAT334_ALERT_2_C Small Aver. Benzene C-C Dist C14      -C19             1.37 Ang.
PLAT342_ALERT_3_C Low Bond Precision on  C-C Bonds ...............    0.00931 Ang.
PLAT417_ALERT_2_C Short Inter D-H..H-D       H4       ..H5       .       2.14 Ang.
                                                   x,1+y,z  =      1_565 Check
PLAT790_ALERT_4_C Centre of Gravity not Within Unit Cell: Resd.  #          1 Note
              C13 H19 Br Co N5 O5
PLAT906_ALERT_3_C Large K Value in the Analysis of Variance ......      3.546 Check
```

Alert level G

```
PLAT007_ALERT_5_G Number of Unrefined Donor-H Atoms ..............          3 Report
PLAT432_ALERT_2_G Short Inter X...Y Contact  O5       ..C18              2.93 Ang.
                                                1+x,-1+y,z  =      1_645 Check
PLAT434_ALERT_2_G Short Inter HL..HL Contact I1       ..Br1              3.42 Ang.
                                                2-x,2-y,-z  =      3_775 Check
PLAT883_ALERT_1_G No Info/Value for _atom_sites_solution_primary .     Please Do !
PLAT941_ALERT_3_G Average HKL Measurement Multiplicity ...........        3.6 Low
PLAT978_ALERT_2_G Number C-C Bonds with Positive Residual Density.          0 Info
```

---

0 **ALERT level A** = Most likely a serious problem - resolve or explain
2 **ALERT level B** = A potentially serious problem, consider carefully
8 **ALERT level C** = Check. Ensure it is not caused by an omission or oversight
6 **ALERT level G** = General information/check it is not something unexpected

1 ALERT type 1 CIF construction/syntax error, inconsistent or missing data
7 ALERT type 2 Indicator that the structure model may be wrong or deficient
6 ALERT type 3 Indicator that the structure quality may be low
1 ALERT type 4 Improvement, methodology, query or suggestion
1 ALERT type 5 Informative message, check

It is advisable to attempt to resolve as many as possible of the alerts in all categories. Often the minor alerts point to easily fixed oversights, errors and omissions in your CIF or refinement strategy, so attention to these fine details can be worthwhile. In order to resolve some of the more serious problems it may be necessary to carry out additional measurements or structure refinements. However, the purpose of your study may justify the reported deviations and the more serious of these should normally be commented upon in the discussion or experimental section of a paper or in the "special_details" fields of the CIF. checkCIF was carefully designed to identify outliers and unusual parameters, but every test has its limitations and alerts that are not important in a particular case may appear. Conversely, the absence of alerts does not guarantee there are no aspects of the results needing attention. It is up to the individual to critically assess their own results and, if necessary, seek expert advice.

Publication of your CIF in IUCr journals

A basic structural check has been run on your CIF. These basic checks will be run on all CIFs submitted for publication in IUCr journals (*Acta Crystallographica*, *Journal of Applied Crystallography*, *Journal of Synchrotron Radiation*); however, if you intend to submit to *Acta Crystallographica Section C* or *E* or *IUCrData*, you should make sure that full publication checks are run on the final version of your CIF prior to submission.

Publication of your CIF in other journals

Please refer to the *Notes for Authors* of the relevant journal for any special instructions relating to CIF submission.

PLATON version of 22/04/2020; check.def file version of 09/03/2020

Datablock III14tfib - ellipsoid plot

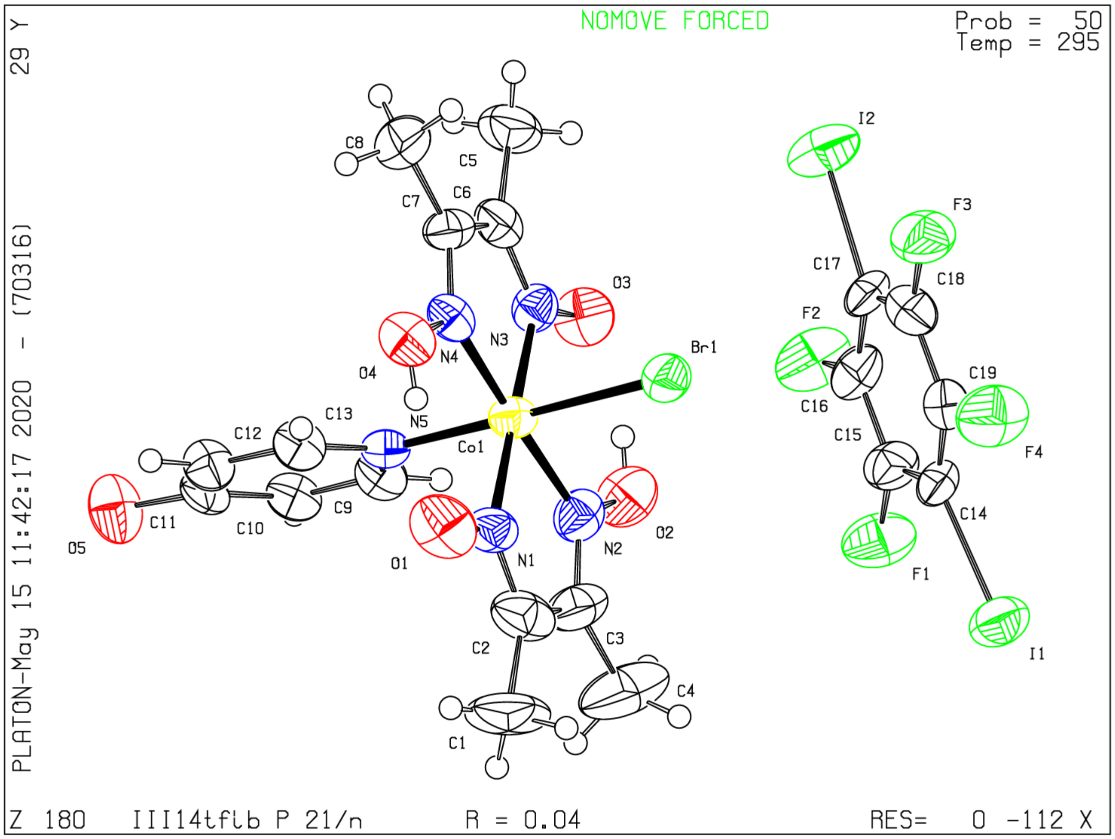

Supplement: Supplementary file 1 [file Checkcif_Bedekovic et. al..pdf › materials_804374- supplement-final.docx/Checkcif_Bedekovic et. al..pdf]
